# Team training in the real world: A cluster-randomized hybrid effectiveness-implementation trial of TeamTRACS in rural Children’s Advocacy Centers

**DOI:** 10.1017/cts.2025.10203

**Published:** 2025-11-26

**Authors:** Elizabeth A. McGuier, Jaely D. Wright, Greg Flett, Scott D. Rothenberger, Eduardo Salas, David J. Kolko

**Affiliations:** 1 Department of Psychiatry, University of Pittsburgh School of Medicine, Pittsburgh, PA, USA; 2 Western Psychiatric Hospital, University of Pittsburgh Medical Center, Pittsburgh, PA, USA; 3 National Children’s Advocacy Center, Huntsville, AL, USA; 4 Department of Internal Medicine, University of Pittsburgh School of Medicine, Pittsburgh, PA, USA; 5 Department of Psychological Sciences, Rice University, Houston, TX, USA

**Keywords:** Teamwork, implementation, team training, hybrid trial, mixed methods

## Abstract

**Introduction:**

Children’s Advocacy Centers (CACs) use multidisciplinary teams to respond to child abuse allegations. These fluid teams can benefit from team training to enhance team functioning and performance and strengthen the workforce, but they need guidance and resources to support the implementation of team training.

**Methods:**

We conducted a cluster-randomized hybrid effectiveness-implementation trial to test the effectiveness of team training and evaluate a self-guided implementation process. Six rural CACs (*N* = 172 team members) were randomized to TeamTRACS (Team Training in Roles, Awareness, Communication, & Support; *n* = 4) or a waitlist comparison (*n* = 2). Simultaneous mixed methods evaluated the effectiveness of TeamTRACS (QUAN + qual) and the implementation process (quan + QUAL).

**Results:**

Reactions to TeamTRACS were positive (mean ratings > 4.5 on 1–5 scale), and TeamTRACS significantly increased teamwork knowledge (estimated marginal means = 80% vs. 75% [intent-to-treat]; 85% vs. 76% [training attendance]). There were no effects on skill use or work-related outcomes. Changes in team-level outcomes were small and inconsistent; one TeamTRACS team made substantial improvements. Reactions to self-guided implementation were positive (mean ratings > 4 on 1–5 scale). However, only one team completed the implementation process. Challenges included difficulty forming and maintaining a change team, turnover and understaffing, and competing priorities and a short timeframe.

**Conclusions:**

Overall, TeamTRACS and its self-guided implementation process were positively received. Incomplete implementation may have limited TeamTRACS’ effectiveness. Longer timeframes and external support may improve the implementation of team training in low-resource settings.

## Background

Children’s Advocacy Centers (CACs) use multidisciplinary teams to provide coordinated interagency responses to allegations of sexual abuse and other severe maltreatment [1–3]. There are >950 CACs in the United States, half in rural areas, and they serve hundreds of thousands of children annually [1]. Teams include CAC staff and professionals employed by independent organizations (e.g., child welfare, law enforcement, victim advocacy, mental health) [2–4]. Teams are fluid, with members participating to varying degrees at varying times depending on their role and involvement in specific cases [5,6]. Effective teamwork is required for these fluid, cross-boundary teams to investigate allegations and connect families with needed services [2].

Teamwork is a broad term referring to both team functioning (i.e., how teams think, feel, and act) and performance. Team functioning includes affective, behavioral, and cognitive processes and states (e.g., trust, communication, shared goals) [7–9], while performance reflects team output, including work quality (e.g., task completion, efficiency, productivity) [7,8]. More than a century of research across diverse work settings has established that team functioning influences performance [8]. In healthcare and child welfare settings, better team functioning and performance are associated with better outcomes for those served by the team [10,11]. In CAC teams specifically, team functioning is positively associated with team member-rated performance [12] and case outcomes (e.g., service receipt) [4]. Better team functioning is also associated with lower burnout and turnover intentions [4,13,14], making it important for the well-being of the child abuse workforce.

Team development interventions can improve team functioning, team performance, and workforce outcomes [15–18]. Team training is a specific type of intervention that targets team functioning by teaching teamwork knowledge, skills, and abilities [16–18]. On average, it has large positive effects on team functioning and performance [15,17]. In healthcare settings, a meta-analysis found that team training results in increased learning, increased skill use, and improved organizational and patient outcomes [16]. However, most team training studies have focused on traditional “fixed” teams with clearly bounded stable membership, and less is known about team training in the context of fluid cross-boundary teams like CAC teams [5,6]. Fluid teams rely more on informal communication, require more intentional coordination, and face greater challenges in negotiating overlapping roles and responsibilities [6]. Team interventions designed for fluid cross-boundary teams are a promising approach to enhancing CAC team functioning, improving team performance, and strengthening the child abuse workforce.

Implementation of team interventions can be challenging, with common barriers including insufficient organizational support, the time and training needed for facilitators, time constraints for participants, and challenges in providing ongoing support and reinforcement of skill use [19,20]. Many team training efforts have been conducted in resource-rich environments (e.g., business, military) and led by researchers and/or individuals with expertise in quality improvement. Because research and quality improvement roles rarely exist in low-resource settings, extending team training to other settings, like rural CACs, requires developing implementation strategies that rely less on this type of expertise and are appropriate for settings with limited resources and quality improvement infrastructure. Strategies to support the use of team training in low-resource settings can extend the reach and impact of team training efforts.

### TeamTRACS: A team training adapted for CAC teams

In a prior study, we adapted TeamSTEPPS, an evidence-based team training intervention, to fit the needs, resources, and context of CAC multidisciplinary teams [21]. TeamSTEPPS is a widely used team training for healthcare settings developed by the Agency for Healthcare Research and Quality [22]. It targets teamwork knowledge, skills, and attitudes, and it has been shown to improve team functioning and patient outcomes [23–25]. Our adapted training, TeamTRACS (**Team T**raining in **R**oles, **A**wareness, **C**ommunication, and **S**upport), targets knowledge, skills, and attitudes related to team member roles, shared awareness, communication, mutual support, and goal-setting. Adaptations included changes to the setting and population, how the training was delivered and evaluated, and training content (e.g., adding information about roles on CAC teams). The adaptation process and modifications are described elsewhere [21].

A mixed methods pilot study of TeamTRACS with one CAC team found that TeamTRACS was acceptable, appropriate, and feasible, and participants rated the training content positively [21]. Participants recommended adding additional structured support before and after the workshop to support the team in taking actions to maximize the training’s impact [21].

### Current study

We conducted a cluster-randomized controlled hybrid effectiveness-implementation trial of TeamTRACS using simultaneous mixed methods. The study had two goals:

Aim 1: Test the effectiveness of TeamTRACS.

Aim 2: Evaluate the acceptability, appropriateness, and feasibility of a structured, self-guided approach to implementation.

**Figure 1.**
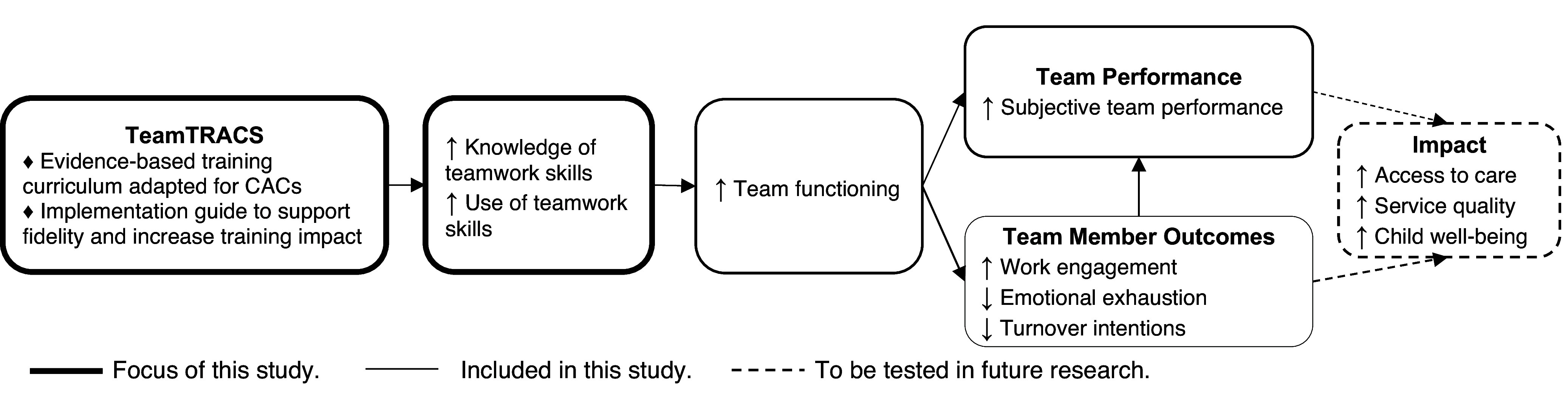
Hypothesized outcomes and impact of TeamTRACS.

For our first aim (QUAN + qual), we evaluated reactions, learning, transfer, and results, aligning with Kirkpatrick’s four-level evaluation framework [26]. We tested differences in teamwork knowledge (*learning*) and skill use (*transfer*). We also explored the effect of TeamTRACS on team functioning, team performance, and individuals’ work-related outcomes (*results*). TeamTRACS was hypothesized to improve teamwork knowledge, use of teamwork skills, and team functioning, therefore improving team performance, workforce outcomes, and ultimately, the quality of existing services and outcomes for children who experience maltreatment (Figure 1). For our second aim, we evaluated the acceptability, feasibility, and appropriateness of a self-guided implementation approach. We also described implementation progress and identified facilitators and barriers to self-guided implementation. This trial was registered on clinicaltrials.gov (NCT05370157) and reported in accordance with reporting guidelines (see supplementary material 1).

## Methods

### Community engagement

We partnered with the National Children’s Advocacy Center (NCAC) to refine our implementation guide, recruit CACs to participate in the trial, and deliver TeamTRACS. NCAC provides training and technical assistance and is a trusted resource for CACs. In addition, a community advisory committee of individuals with expertise in CACs provided feedback on research questions, study methods, interpretation of results, and dissemination plans [21].

### Site recruitment

We recruited six rural CACs from the southern United States. The sample size was determined by budgetary constraints. The NCAC advertised the study through emails to CACs, and the research team and NCAC co-hosted an online information session. Interested CACs were directed to discuss their study with their team and then submit a brief survey with basic information about their team (e.g., location, team size) and why they were interested in the study. Participating CACs received $250 to recognize the administrative time needed to support participation.

### Participants

Participants were multidisciplinary team members at participating CACs. Only current team members were eligible to participate at each timepoint (see Supplemental Figure 1 in supplementary material 2). CAC staff identified team members before each assessment.

### Procedures

#### Randomization

CACs were randomized to TeamTRACS (*n* = 4) or a waitlist comparison (*n* = 2) after baseline data collection. We aimed to balance team size across conditions by creating three pairs of CACs that were as equal in number of participants as possible and then randomizing pairs to condition using a random number generator.

#### Assessment procedures

Quantitative data were collected through online surveys at baseline (prior to randomization) and follow-up (7 months after baseline; 2–3 months after the TeamTRACS workshop) and brief post-training surveys. Baseline and follow-up surveys were constructed in REDCap, a secure web-based platform, and individual survey invitations were emailed to all team members. Participants received $20 for each completed survey. Brief post-workshop surveys (<15 minutes) were completed on paper for in-person training workshops and through REDCap for virtual workshops.

For teams randomized to TeamTRACS, we conducted periodic reflections on the implementation process with a key informant in each CAC [27]. Reflections were conducted via videoconference approximately once a month. Participants received $30 for each reflection. The University of Pittsburgh Institutional Review Board approved all procedures. All participants provided informed consent.

#### Intervention conditions

##### TeamTRACS Intervention and Implementation Guide

CACs randomized to the intervention condition were offered TeamTRACS facilitated by an experienced team trainer from NCAC who used standard presentation slides and a detailed instructor guide. Each module includes didactic instruction, discussion, and interactive activities (e.g., identifying team strengths/weaknesses, role plays of new skills). Training materials are open source and available from the first author. The workshop could be delivered in-person or virtually; teams chose the delivery method and length (4–8 hours) as part of the implementation process. Intervention CACs also received a companion implementation guide to support their team in implementing and maximizing the effectiveness of TeamTRACS. The guide was designed to facilitate a self-guided implementation approach that requires few resources and supports teams in taking actions to improve teamwork. The implementation guide was adapted from TeamSTEPPS implementation materials and structured in four phases based on the EPIS (Exploration, Preparation, Implementation, and Sustainment) framework [28]. It incorporates principles of effective team training [29] as well as feedback from our pilot study [21], our community advisory committee, and NCAC staff.

The 10-step guide integrates multiple discrete implementation strategies [30], including organize implementation teams (Step 1), assess for readiness and identify barriers and facilitators (Steps 1–3), develop an implementation blueprint (Step 4), and develop and implement tools for quality monitoring (Steps 6–8). Each specific step includes key actions, tools and resources, tips for success, and questions to answer before moving to the next step. The guide also includes suggestions for managing common challenges (e.g., preparing for turnover). Activities were designed to be completed by “change team” members at each CAC.

#### Waitlist

CACs randomized to the waitlist condition were offered teamTRACS after completion of follow-up data collection. The same trainer facilitated all training workshops.

### Measures: Evaluation of TeamTRACS

#### Background information

As part of the baseline and follow-up surveys, participants reported their discipline, years working with their team, gender, race, ethnicity, and age. CAC staff provided data on attendance at monthly team case review meetings and changes in team members during the study. The trainer recorded attendance at the training workshops.

#### Reactions to training

Immediately after completing each TeamTRACS module, participants were asked to complete a brief survey. They rated the module’s relevance and usefulness on a 5-point Likert scale (1 “*completely disagree*” to 5 “*completely agree*”) and the likelihood of using the skills and strategies from the module on a 5-point scale (1 “*not at all likely*” to 5 “e*xtremely likely*”). Participants also provided open-ended comments on what skills they plan to use, what they found most useful, what they found most challenging, and suggestions for improvement. After completing all modules, participants rated overall relevance, usefulness, and likelihood of using skills/strategies and provided additional open-ended comments. They also rated the acceptability, appropriateness, and feasibility of the overall training; each outcome was assessed with two items rated on a 5-point scale from 1 “*completely disagree*” to 5 “*completely agree*” [31].

#### Teamwork knowledge

We developed 16 multiple choice questions (3–4 per module) to assess knowledge taught in the TeamTRACS workshop (e.g., “What is a check-back?”). Item development was informed by TeamSTEPPS knowledge assessments [32,33]. Knowledge was assessed at baseline and follow-up for all participants. Workshop participants also completed knowledge questions as part of the brief survey following each module. Scores were calculated as a percentage of correct responses (possible range 0–100%).

#### Use of teamwork skills

We developed 41 items reflecting specific teamwork skills taught in TeamTRACS (see Table 1). Most items were adapted from existing scales [34–37]. Team members rated how frequently they used each skill. Internal consistency was high (*α* = 0.96 at both timepoints). Mean total scores were used in analyses.

**Table 1.** Rating scales and sample items for study measures

Domain	Number of items	Rating scale	Sample item
**Teamwork skill use**	41		
Team structure & roles	5	1 “*never*” to 5 “*almost always*”	I identify and describe my abilities and contributions to the team.
Shared awareness	7		I pay attention to how other team members are doing (e.g., workload, stress level).
Communication: information exchange	12		I allow enough time for questions when communicating with others.
Communication: assertion	6		I raise concerns in a way that is respectful of other team members.
Mutual support	7		I acknowledge the hard work of other team members.
Reflection & goal-setting	4		I discuss with other members how the team is working.
**Team functioning**			
*Team structure & roles*			
Understanding and respect of team structure & roles	5	1 “*strongly disagree*” to 5 “*strongly agree*”	I respect the disciplines represented on our team.
*Shared awareness*			
Shared awareness	6	1 “*strongly disagree*” to 5 “*strongly agree*”	Team members are aware of progress toward goals.
*Communication*			
Information exchange	4	1 “*strongly disagree*” to 5 “*strongly agree*”	The members of this team carefully consider the unique information provided by each individual team member.
Conflict management	5	1 “*not at all*” to 5 “*to a very great extent*”	To what extent does your team actively work to encourage healthy debate and exchange of ideas?
Psychological safety	7	1 “*very inaccurate*” to 7 “*very accurate*”	Members of this team are able to bring up problems and tough issues.
*Mutual support*			
Supportive behavior	5	1 “*not at all true*” to 5 “*totally true*”	We encourage each other to do a good job.
*Reflection & goal-setting*			
Reflexivity	4	1 “*strongly disagree*” to 5 “*strongly agree*”	We regularly discuss whether the team is working effectively.
Learning behavior	7	1 “*very inaccurate*” to 7 “*very accurate*”	This team frequently seeks new information that leads us to make important changes.
Clear direction	3	1 “*very inaccurate*” to 7 “*very accurate*”	It is clear what this team is supposed to accomplish.
**Team performance**			
Overall performance	5	1 “*very inaccurate*” to 7 “*very accurate*”	The quality of work done by this multidisciplinary team is improving over time.
**Team member outcomes**			
Emotional exhaustion	9	1 *“a few times a year or less*” to 6 “*every day*”	I feel burned out from my work.
Work engagement	6	1 “*strongly disagree*” to 5 “*strongly agree*”	When working with the team on cases I devote a lot of energy.
Turnover intentions	3	1 “*strongly disagree*” to 5 “*strongly agree*”	I have seriously thought about leaving my organization.

#### Team functioning and performance

We used several measures to assess domains of team functioning targeted by TeamTRACS. Outcomes were understanding and respect of team roles [38,39], shared awareness [35,36], information exchange [40], conflict management [41], psychological safety [42], supportive behavior [43], reflexivity [37], learning behavior [42], and shared goals [42]. Subjective team performance was assessed with a validated scale [42]. Table 1 shows sample items and how these measures align with TeamTRACS modules. All measures had good internal consistency (*α*’s = 0.79–0.94). See Supplemental Table 1 in supplementary material 2 for psychometric information.

#### Team member outcomes

We assessed three individual-level work-related outcomes: emotional exhaustion, work engagement, and turnover intentions. Emotional exhaustion was assessed with a subscale of the Maslach Burnout Inventory [44]. Work engagement was assessed with six items [45]. Lastly, participants rated their intentions to leave their current job [46]. See Table 1 for sample items and Supplemental Table 1 in supplementary material 2 for psychometric information.

### Measures: Evaluation of self-guided implementation

#### Reactions to self-guided implementation

A key informant in each CAC rated the acceptability, appropriateness, and feasibility of self-guided implementation at the beginning, middle, and end of the study. Each outcome was assessed with two items [31]. Items were highly intercorrelated (*r* = 0.58–1.00) and combined by averaging ratings at each timepoint. Internal consistency was excellent at all timepoints (*α*’s = 0.94–0.96).

#### Periodic reflections and document collection

Reflections with key informants provided detailed information on the implementation process as it occurred, including steps taken, challenges, and future plans [27]. We tracked the completion of implementation activities to assess fidelity to the implementation guide. Informants also provided feedback on the implementation guide and suggestions for improvement. We collected copies of relevant documents (e.g., completed worksheets) throughout the implementation process.

### Data analyses

#### Preparatory analyses

For quantitative data, we calculated survey response rates, looked for patterns of missing data, and examined descriptive statistics. We examined within-team agreement on measures of team functioning and performance using the Average Deviation index (AD_md_). Lower scores indicate better agreement, and scores below recommended upper limits indicate sufficient agreement within teams to justify aggregation [47].

Our measures of psychological safety, learning behavior, and overall performance exhibited poor team-level agreement in this sample, with AD_md_ values above the recommended upper limit for one or more teams at each timepoint (Supplemental Table 1 in supplementary material 2). Accordingly, we analyzed these measures as individual-level outcomes instead of creating aggregate team scores. For measures with adequate agreement, we created aggregate scores for each team reflecting the mean of team member scores.

#### Effectiveness of TeamTRACS (QUAN + qual)

We examined descriptive statistics for acceptability, appropriateness, and feasibility ratings of TeamTRACS. We also examined descriptive statistics for post-workshop ratings of relevance, usefulness, and likelihood of using skills and reviewed open-ended comments about the training. To evaluate the effects of TeamTRACS on team members’ knowledge and skill use, we conducted intent-to-treat analyses testing changes in knowledge and skill use from baseline to follow-up using linear mixed models to account for repeated measures and clustering within teams. We also conducted analyses comparing participants who attended the training to those who did not attend it. Analyses were conducted in R using the *lme4* package and restricted maximum likelihood estimation (REML). Models included fixed effects of time (baseline vs. follow-up), intervention condition, and their interaction as well as random effects for person and team. We used the *clubSandwich* package to calculate cluster robust standard errors.

We also explored the effects of TeamTRACS on team functioning, team performance, and individuals’ work-related outcomes. These measures included both individual-level outcomes (i.e., psychological safety, learning behavior, team performance, work engagement, emotional exhaustion, turnover intentions) and team-level outcomes (i.e., team roles and respect, shared awareness, information exchange, conflict management, supportive behavior, reflexivity, clear direction). Changes in individual-level outcomes were tested using linear mixed models as described above. Because this study was underpowered for team-level outcomes, we plotted data for team-level measures of team functioning and performance to visually examine team-level scores at baseline and follow-up and did not conduct hypothesis testing.

#### Evaluation of self-guided implementation (quan + QUAL)

The second goal of this study was to evaluate a self-guided approach to implementing TeamTRACS. We examined descriptive statistics for acceptability, appropriateness, and feasibility ratings of self-guided implementation. We assessed the proportion of implementation activities completed in each intervention CAC as a measure of fidelity to the implementation guide.

We used rapid analysis [48] to create summaries of periodic reflections. Interviewers reviewed automatically generated interview transcripts and created summaries using a spreadsheet template. The template listed multiple domains (e.g., implementation progress, positive feedback, suggestions for change) and provided space for key points and exemplar quotes in each domain. At the end of the study, we integrated summaries from individual interviews into an overarching narrative of implementation for each team. Then, we compared and contrasted narratives across teams to identify themes, including facilitators and barriers to self-guided implementation.

## Results

### CAC characteristics

Participating CACs were in five states in the southern region of the United States. Twelve CACs expressed interest, seven CACs were invited to participate, and six CACs agreed. Of the CACs that were not invited to participate, two were in urban areas, two were participating in other team improvement projects, and one served multiple urban and rural counties with combined case review meetings. All participating teams reported serving rural populations; county populations ranged from approximately 36,000 to 56,000.

### Participation rates

Table 2 shows the number of survey responses and participation rates by team and overall. As expected, team members changed during the study; there were 172 unique participants across timepoints (Supplemental Figure 1 in supplementary material 2). Key informants were the team coordinator at each CAC; some also had another role (e.g., advocate, forensic interviewer). For 3 of 4 teams, one key informant completed all periodic reflections and ratings of the self-guided implementation process. In the remaining team, the key informant changed partway through the study because the initial informant went on leave.

**Table 2.** Study participation and training workshop format

	Team size		Survey participation		Number with baseline & follow-up data		
	*Baseline*	*Follow-up*	*Baseline*	*Follow-up*		Workshop format	Workshop attendees
TeamTRACS team 1	23	26	18 (78%)	24 (92%)	16	In-person 6 hours	13
TeamTRACS team 2	28	29	20 (71%)	22 (76%)	12	Virtual 4 hours	13
TeamTRACS team 3	37	40	24 (65%)	21 (55%)	13	In-person 6 hours	15
TeamTRACS team 4	42	41	25 (60%)	22 (54%)	18	In-person 4 hours	12
Waitlist team 1	21	21	16 (76%)	12 (57%)	10	–	–
Waitlist team 2	46	45	32 (70%)	28 (62%)	23	–	–
*Total*	*197*	*202*	*135 (69%)*	*129 (64%)*	*92*		

### Participant characteristics

Participant characteristics were assessed using baseline responses when available and follow-up responses for those without baseline responses. Participants identified as non-Hispanic white (73%), Hispanic/Latino of any race (7%), American Indian/Alaska Native (6%), Black/African American (5%), and Asian (1%); the remainder (8%) were missing or selected “prefer not to answer.” Participants identified as female (66%) and male (27%); the remainder (7%) selected “prefer not to answer.” Supplemental Figure 2 in supplementary material 2 shows participants’ professional disciplines. Most participants had worked with their team for more than 1 year (13% <1 year; 33% 1–3 years; 23% 4–6 years; 32% 7 years or more).

### Training delivery

Table 2 shows the workshop format and length chosen by each participating team. In-person training workshops were conducted in a single location chosen by the team, and the virtual training was delivered through Zoom with participants joining individually from locations of their choosing. Because teams determined who to invite, the proportion of invited members who participated is unknown. All workshops were facilitated by the same experienced team trainer using the same materials (i.e., training slides, instructor guide). Although workshop timing and pace varied, all content was delivered during each workshop. Some teams provided food or other incentives for team members to attend; the research team did not provide any incentives.

### Reactions to training

Most training attendees completed ratings of the workshop content (*n* = 41; 77%). Overall, training attendees rated the content highly relevant (*M* = 4.83) and useful (*M* = 4.83) and reported that they were very to extremely likely to use the skills taught (*M* = 4.59). Ratings for individual modules were similarly high. Attendees agreed that the training was acceptable (*M* = 4.63, range 4–5), appropriate (*M* = 4.70, range 4–5), and feasible (*M* = 4.54, range 3–5). Open-ended comments were positive, with some attendees suggesting that the training should be longer.

### Descriptive data

Supplemental Table 2 in supplementary material 2 provides descriptive statistics and intercorrelations for baseline measures of teamwork knowledge and skill use, team functioning, team performance, and team member outcomes. Most measures were correlated with one another, except for teamwork knowledge scores which were not significantly correlated with any other measures.

### Changes in teamwork knowledge

**Figure 2.**
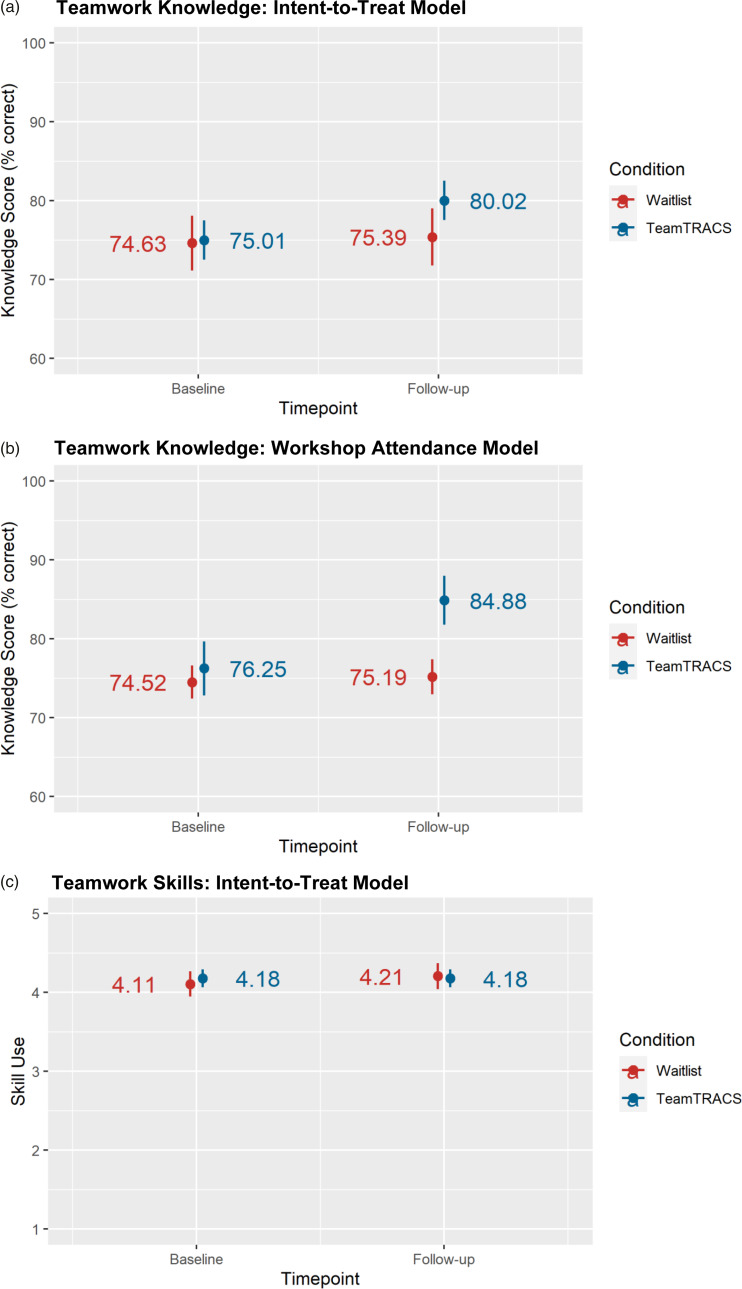
Teamwork knowledge and skills: Marginal effects of intervention condition & timepoint. *Note:* (a) Teamwork knowledge: Intent-to-treat model. (b) Teamwork knowledge: Workshop attendance model. (c) Teamwork skills: Intent-to-treat model.

In an intent-to-treat mixed model (*N* = 165), there was a significant interaction of condition and time; participants in teams randomized to TeamTRACS exhibited higher knowledge scores at follow-up than participants in waitlist teams (Table 3, Model 1; Figure 2a). The same interaction was found when comparing participants who attended a TeamTRACS workshop to those that did not attend (Table 3, Model 2; Figure 2b). Knowledge scores for those who attended a TeamTRACS workshop were high immediately after the workshop (average score = 90%). These results indicate that TeamTRACS was effective in increasing teamwork knowledge.

**Table 3.** Changes in teamwork knowledge and skill use by intervention condition and training workshop attendance

	Knowledge			Skill use		
	Conditional ICC	Unstandardized coefficient (SE)	95% CI	Conditional ICC	Unstandardized coefficient (SE)	95% CI
*Model 1: Intent-to-treat*	0.306			0.753		
TeamTRACS condition		0.38 (2.26)	−8.81–9.57		0.07 (0.14)	−0.50–0.65
Time		0.77 (0.48)	−5.26–6.80		0.10 (0.02)	−0.19–0.40
Condition*Time		4.25* (0.85)	0.76–7.74		−0.10 (0.08)	−0.40–0.20
*Model 2: Workshop attendance*	0.279			0.752		
TeamTRACS workshop attendance		1.73 (1.89)	−3.64–7.10		0.04 (0.09)	−0.22–0.30
Time		0.67 (0.83)	−1.63–2.98		0.04 (0.06)	−0.12–0.20
Attendance*Time		7.96* (1.95)	2.58–13.33		−0.02 (0.07)	−0.21–0.17

* *p* < 0.05.

### Changes in teamwork skill use

Use of teamwork skills was high at both timepoints (baseline *M* = 4.17, follow-up *M* = 4.19). Both intent-to-treat and workshop attendance mixed models found no significant effects of condition, time, or their interaction on skill use (Table 3; Figure 2c).

### Exploratory analyses: Changes in team functioning and performance

#### Individuals’ perceptions of team outcomes

Intent-to-treat linear mixed models found no significant effects of condition, time, or their interaction on individuals’ perceptions of psychological safety, learning behavior, or team performance. See Supplemental Figures 3–5 in supplementary material 2 for plots of marginal effects.

#### Team-level outcomes

Plots of team-level outcomes by condition and timepoint are presented in Supplemental Figures 6–12 in supplementary material 2. Most teams exhibited small and inconsistent changes over time. One intervention team (TeamTRACS Team 3) exhibited increases greater than 0.35 on 6 of 7 measures, indicating substantial improvement in several outcomes. The largest increases were in reflexivity (mean difference = 0.85) and clear direction (mean difference = 1.12). This team had the lowest baseline scores on 6 of 7 measures, indicating poorer baseline functioning than the other participating teams. No other teams exhibited consistent patterns of change.

### Exploratory analyses: Changes in workforce outcomes

Intent-to-treat linear mixed models found no significant effects of condition, time, or their interaction on individuals’ work engagement, emotional exhaustion, or turnover intentions. Plots of marginal effects are provided in Supplemental Figures 13–15 in supplementary material 2. Individuals in the comparison condition reported lower emotional exhaustion at baseline that increased over time (predicted means = 1.92 baseline and 2.21 follow-up), while individuals in the intervention group were relatively stable (predicted means = 2.30 baseline and 2.21 follow-up); however, this interaction was not significant in mixed models (*p* = .12).

### Evaluation of self-guided implementation

#### Reactions to self-guided implementation

Key informants provided ratings of the acceptability, appropriateness, and feasibility of self-guided implementation. Ratings were positive, with average scores greater than 4 (“agree”) at the beginning, middle, and end of the study (*M* = 4.17, *M* = 4.17, *M* = 4.29, respectively). Participants provided substantial feedback during reflections and appeared comfortable reflecting on their progress. Participants appreciated the structure of the guide and provision of worksheets (see Table 4).

**Table 4.** Qualitative findings: Facilitators and barriers to implementation of TeamTRACS

Theme	Quotes
Guide was structured and easy to follow.	“I thought it was really great. It was super easy to follow, it laid everything out for us really well.” (TeamTRACS Team 2) “It’s extremely organized. I consider myself an organized person, so I appreciated that. And I mean, like the worksheets that went along with the guide, are super helpful and makes it easy for somebody to just get a hold of the material and be able to go through like A B and C with the team. So it makes it very easy to implement” (TeamTRACS Team 3) “I think it was very helpful in guiding the conversations we were having. It gave me the start of the topics that we should talk about. I think we went much further.” (TeamTRACS Team 4) “I like the worksheets. The way it broke it down who does what, you know, specific, measurable. And then you answer them all. And then you redefine the smart version of your goal from your initial. I think you broke it down really well, because we identified what we were going to do, who was going to do it. And what the responsibilities are, starting and end dates, and even listed out different tools and stuff that we might use, stuff that I probably wouldn’t have thought of if it hadn’t been on here in a block.” (TeamTRACS Team 1) “I really like [the worksheets]. That’s actually my favorite part. I like knowing what I need to do and when I need to do it.” (TeamTRACS Team 2)
Difficulty forming and maintaining a change team.	“I have not been able to get my team back together…and so I am a solo team.” (TeamTRACS Team 4) “I just really came to the realization that I really am the sole person who’s moving this study forward. I’m not really getting help from any other partner about getting buy-in from other members.” (TeamTRACS Team 3) “They kind of feel like it’s our job to implement it and they don’t want to be part of it, but I can’t implement something by myself.” (TeamTRACS Team 4)
Frequent turnover and understaffing.	“In one of our agencies they had a complete mix up of admin, like literally just shook the bag and reassigned jobs to everybody. …because of some of that shake up in the agencies, there’s been a lot of lack of knowledge of what we’re trying to do, and therefore lack of acceptance…it’s hard to get people on board to change things if they do not even know what they’re doing.” (TeamTRACS Team 4) “We have so much turnover [in child welfare] that I’ve stopped learning their names until they’ve been there at least six months. Because they’re just in and out and in and out so we do not have any real consistency.” (TeamTRACS Team 2) “We’ve had a lot of turnover. We have not had anybody stay for longer than 6 weeks with our CPS [child protective services].” (TeamTRACS Team 4)
Competing priorities and short timeframe.	“[Implementing TeamTRACS] hasn’t been a top priority as of right now…but it always relates directly to my job. …I would say day to day it’s not a number one task, but it is something that’s always on the back burner…I would say that it’s a part of my job, but not a part of my day-to-day work.” (TeamTRACS Team 3) “I’d say out of the top 10, 1 being the most important, I’d say it is probably down to like a 5 around the holidays just because we have so much extra coming in on us, and only 3 of us here. We got an interview coming in and I had to push it. …before that, it was probably like 2 or 3. We kept it up and moving, and [then] we sort of pushed it aside because we had to get the Christmas stuff done and had to get it out the door while still doing interviews.” (TeamTRACS Team 1) “Sometimes we get crazy, and I wear multiple hats, so probably it’s not our number one priority. But it’s probably in the middle somewhere.” (TeamTRACS Team 4)

Quotes were lightly edited for clarity.

#### Progress through self-guided implementation

Only one team completed all steps in the implementation guide. Some teams continued to progress through the implementation guide after the end of the study and completion of the follow-up survey. See supplementary material 2 in supplementary material 2 for a table depicting the progress each team made through self-guided implementation and summaries of implementation progress in each team based on the findings from periodic reflections. Most teams did not complete later implementation steps intended to encourage teamwork skill use, which may have limited the impact of TeamTRACS on skill use and team outcomes.

#### Challenges to self-guided implementation

Most teams (3 of 4) struggled to form and maintain a change team. The only team to complete all implementation steps was the team that successfully created a change team, suggesting that early and consistent engagement of a change team facilitates implementation progress. Without a change team, responsibility for implementation typically fell to one CAC staff member (see Table 4).

All teams experienced frequent turnover and understaffing that affected their engagement in implementation as well as attendance at the training workshop. In 2 of 4 teams, the primary contact person and implementation lead was promoted during the study. In one team, this person continued to lead implementation efforts, allowing for continued progress, while in the second team responsibility was passed to another person and progress slowed. All teams reported that attendance at the training workshop was limited because of emergent demands on team members. For example, in one team, all child welfare workers were unable to attend the training workshop because they were required to provide coverage for a neighboring county with severe understaffing.

Participants also identified competing priorities that made implementation challenging, particularly within the short timeframe of the study. Key informants described TeamTRACS implementation and strengthening team functioning as an important part of their job but not part of their daily tasks (see Table 4). Informants also reported holiday-related demands (e.g., events, vacations) that slowed implementation progress after the training workshops were conducted. For some teams, qualitative data suggested continued progress and improvements after the follow-up survey was completed.

## Discussion

This study tested the effectiveness of TeamTRACS, a team training intervention for CAC multidisciplinary teams, and evaluated its implementation. It contributes to existing scientific literature by examining the effectiveness of team training in fluid cross-boundary teams [5] and extending outside of hospital and healthcare settings where most research on interprofessional collaboration has been conducted. It also provides insight into how team training is implemented in real-world settings.

Our evaluation of TeamTRACS effectiveness was grounded in Kirkpatrick’s framework [16,26]. We evaluated reactions to training, learning (i.e., knowledge change), transfer (i.e., change in skill use), and results (i.e., changes in team functioning and performance). Reactions to TeamTRACS were very positive, and there were significant increases in knowledge for participants in the TeamTRACS condition.

Self-reported skill use was high and did not differ over time or by condition. All participating teams sought out the training and described challenges in the domains targeted by the training, suggesting a need for improvement despite high ratings of skill use. Our measure of skill use was developed for this study, and because baseline scores were high, there was little room for improvement. Participants’ ratings were likely inflated by self-report biases, including social desirability bias [49]. It is also possible that skill practice during the workshop increased participants’ understanding and awareness of their teamwork skills, leading participants to recalibrate their self-ratings downward following the training and resulting in an underestimate of training effectiveness [49]. Relatively few studies have examined changes in skill use after interprofessional training, and skill use is typically self-reported [50]. Future research could use observational measures, standardized role plays, or ratings of other participants’ skill use to reduce self-report biases and better measure teamwork skill use.

Most changes in team functioning and performance were small and inconsistent, although one TeamTRACS team exhibited substantial and consistent improvements. Our findings suggest that team training can improve teamwork in fluid cross-boundary teams; however, incomplete implementation of TeamTRACS, as discussed below, may have limited its effectiveness. There were no significant effects of TeamTRACS on individuals’ work engagement, emotional exhaustion, or turnover intentions.

Users viewed the implementation guide and self-guided process favorably. However, only one team completed all steps in the implementation guide. Challenges to implementation included difficulty forming and maintaining change teams, turnover and understaffing, competing priorities, and the short timeframe of the study. Turnover is a common barrier to implementation in team-based settings [9] and may be especially relevant for interventions targeting the team. Team training efforts that focus on changing team processes and structures (e.g., revising protocols, adjusting meeting agendas), rather than individual behaviors, may be more effective in settings with high turnover and fluidity. Future studies should also examine ways to assess the extent of team fluidity to better understand its effects.

In this study, training workshops were conducted at the end of the year, prior to holiday events and vacations, and most teams only had a month (January) to make changes before the follow-up survey was conducted. The short timeframe for follow-up may have limited our ability to find changes. Final reflections suggested that some teams continued to progress through the implementation guide after the follow-up survey. In a test of team training in a surgical ward, Aaberg and colleagues [51] were able to complete all implementation steps in a 12-month period. The researchers in that study were actively involved as part of the change team, which may have facilitated progress [51]. We found that periodic reflections likely served as a prompt for greater attention to TeamTRACS implementation as well as attention to team functioning. Future efforts to implement TeamTRACS should consider a longer implementation period and integrating recurring check-ins with someone external to the team to help teams maintain their focus.

The limited impact of TeamTRACS on skill use and team-level outcomes may be at least partially because most teams did not complete later implementation steps intended to encourage skill use. Interestingly, the team that struggled the most to implement TeamTRACS was the only team with consistent improvements in team outcomes from baseline to follow-up. This may be because the team’s baseline team scores were low, and there was more room for improvement. Persistent attention to how the team was working and greater reflexivity may have led to improvements even without completion of all implementation steps. This result is encouraging, as it suggests that even incomplete implementation of team training can lead to improvements in fluid cross-boundary teams. However, these findings may simply reflect regression to the mean and should be interpreted cautiously given the overall null findings for team-level outcomes.

### Limitations and future directions

Although this study found significant improvements in teamwork knowledge, it is unclear how meaningful these changes are, as teamwork knowledge was not correlated with skill use or other outcomes at baseline. Although our study is relatively large compared to other studies of team training, we did not have enough power to empirically test our full theory of change (Figure 1). Similarly, with only six teams, this study was not powered to detect changes in team-level measures of team functioning and performance. Larger studies are needed to test the effects of TeamTRACS on team-level outcomes. Survey participation rates varied by team and timepoint, and non-participants may have perceived the team differently. Periodic reflections were conducted with the same informant in most CACs. Although this allowed for continuity over time, other informants may have had different perspectives on implementation. In addition, there was no attention control in the comparison condition.

Our rigorous mixed methods study contributes to the limited literature on effective strategies to improve teamwork and interprofessional collaboration [52] and extends this work to fluid teams operating outside of healthcare settings [5]. Team trainings such as TeamTRACS are flexible approaches to improving teamwork that can be integrated with other training approaches, such as simulation training, to further enhance their effectiveness. We found that our structured, self-guided implementation approach was feasible and identified facilitators and barriers to implementation progress. This study was conducted in real-world settings using existing training infrastructure, increasing the generalizability of our findings to other settings with limited resources to support teams.

## Conclusions

We tested the effectiveness of TeamTRACS in fluid cross-boundary teams serving children experiencing maltreatment. TeamTRACS was positively received and increased teamwork knowledge, but there were no effects on teamwork skill use or individuals’ work-related outcomes, and team-level changes were inconsistent. We also evaluated a self-guided implementation approach, advancing the development of realistic implementation strategies for high-need, low-resource settings. Self-guided implementation was positively received but challenging for teams to complete within a short timeframe. Additional efforts are needed to build capacity for implementation and continuous improvement in low-resource settings. Interventions to improve teamwork in fluid teams have the potential to improve interprofessional collaboration across systems and ultimately improve service quality and outcomes.

## Supporting information

10.1017/cts.2025.10203.sm001 Mcguier et al. supplementary material 1 Mcguier et al. supplementary material

10.1017/cts.2025.10203.sm002 Mcguier et al. supplementary material 2 Mcguier et al. supplementary material

## Supplementary material

The supplementary material for this article can be found at https://doi.org/10.1017/cts.2025.10203.
