# Supplementary material for: Team training in the real world: A cluster-randomized hybrid effectiveness-implementation trial of TeamTRACS in rural Children’s Advocacy Centers
Source: J Clin Transl Sci. 2025 Nov 26;9(1):e275. doi: 10.1017/cts.2025.10203 (PMC12779490; doi:10.1017/cts.2025.10203)
Supplement: Mcguier et al. supplementary material 2 — Mcguier et al. supplementary material [file S2059866125102033sup002.docx]

**Table of Contents: Supplemental Materials**

Table 1: Psychometric Properties of Study Measures2

Table 2: Descriptive Statistics and Intercorrelations for Baseline Measures 3

Table 3: Progress through the Implementation Guide by Team5

Figure 1: Participant Flow Diagram7

Figure 2: Participant Disciplines 8

Figures 3-5: Plots of Marginal Effects for Individual-Level Team Outcomes 9

Figures 6-12: Plots of Team-level Outcomes 10

Figures 13-15: Plots of Marginal Effects for Individual-Level Workforce Outcomes 14

Summaries of Implementation Progress by Team16

# Table 1

*Psychometric Properties of Study Measures*

| **Domain** | **Cronbach’s alpha** | | **Within-Team** **Agreement**  % of teams below AD_md_ limit | |
| --- | --- | --- | --- | --- |
|  | *Baseline* | *Follow-up* | *Baseline* | *Follow-up* |
| ***Team Functioning*** |  |  |  |  |
| *Team Structure & Roles* |  |  |  |  |
| Understanding and respect of team structure & roles | .89 | .88 | 100% | 100% |
| *Shared Awareness* |  |  |  |  |
| Shared awareness | .94 | .92 | 100% | 100% |
| *Communication* |  |  |  |  |
| Information exchange | .87 | .93 | 100% | 100% |
| Conflict management | .90 | .94 | 100% | 100% |
| Psychological safety ^1^ | .84 | .87 | 83% | 67% |
| *Mutual Support* |  |  |  |  |
| Supportive behavior | .91 | .92 | 100% | 100% |
| *Reflection & Goal-Setting* |  |  |  |  |
| Reflexivity | .94 | .93 | 83% | 83% |
| Learning behavior ^1^ | .84 | .87 | 50% | 83% |
| Clear direction | .84 | .87 | 100% | 83% |
| ***Team Performance*** |  |  |  |  |
| Overall performance ^1^ | .79 | .80 | 50% | 67% |
| ***Team Member Outcomes*** |  |  |  |  |
| Emotional exhaustion | .93 | .93 | NA | NA |
| Work engagement | .87 | .87 | NA | NA |
| Turnover intentions | .92 | .92 | NA | NA |

AD_md_: Average deviation of the median

^1^ Insufficient agreement to justify aggregation to the team level.

**Table 2**

*Descriptive Statistics and Correlations among Individual-Level Measures (Baseline Data; N = 126)*

|  | **M (SD)** | **Range** | **2** | **3** | **4** | **5** | **6** | **7** | **8** | **9** | **10** | **11** | **12** | **13** | **14** | **15** |
| --- | --- | --- | --- | --- | --- | --- | --- | --- | --- | --- | --- | --- | --- | --- | --- | --- |
| **1.** Teamwork Knowledge | 75.29 (10.82) | 37.5-93.75 | .07 | .12 | -.01 | -.01 | -.02 | .03 | -.02 | -.11 | -.06 | .01 | .08 | .02 | -.18 | -.13 |
| **2.** Teamwork Skill Use | 4.17  (0.53) | 1.83- 5.00 |  | .55** | .59** | .52** | .46** | .50** | .49** | .58** | .57** | .66** | .30* | .59** | -.09 | -.17 |
| **3.** Team Structure & Roles | 4.40  (0.57) | 2.20- 5.00 |  |  | .69** | .65** | .51** | .50** | .49** | .42** | .45** | .49** | .39** | .51** | -.34* | -.32* |
| **4.** Shared Awareness | 3.92 (0.71) | 2.00- 5.00 |  |  |  | .81** | .70** | .60** | .67** | .67** | .70** | .72** | .43** | .47** | -.30* | -.34* |
| **5.** Information Exchange | 4.10 (0.70) | 2.25- 5.00 |  |  |  |  | .81** | .75** | .72** | .74** | .71** | .73** | .52** | .42** | -.28* | -.21 |
| **6.** Conflict Management | 3.95 (0.72) | 1.80-  5.00 |  |  |  |  |  | .73** | .80** | .69** | .72** | .72** | .48** | .32* | -.28* | -.21 |
| **7.** Psychological Safety | 5.51  (1.09) | 2.86- 7.00 |  |  |  |  |  |  | .71** | .55** | .66** | .70** | .71** | .44** | -.44** | -.34* |
| **8.** Supportive Behavior | 4.25 (0.67) | 2.40- 5.00 |  |  |  |  |  |  |  | .60** | .69** | .75** | .47** | .50** | -.40** | -.31* |
| **9.** Reflexivity | 3.61 (0.97) | 1.25- 5.00 |  |  |  |  |  |  |  |  | .79** | .68** | .34* | .30* | -.19 | -.19 |
| **10.** Learning Behavior | 4.84  (1.02) | 2.29- 7.00 |  |  |  |  |  |  |  |  |  | .75** | .35* | .38** | -.26 | -.24 |
| **11.** Clear Direction | 5.69  (1.23) | 2.33- 7.00 |  |  |  |  |  |  |  |  |  |  | .47** | .50** | -.29* | -.22 |
| **12.** Overall Performance | 5.57  (1.22) | 2.20- 7.00 |  |  |  |  |  |  |  |  |  |  |  | .22 | -.48** | -.34* |
| **13.** Work Engagement | 4.29  (0.56) | 3.00- 5.00 |  |  |  |  |  |  |  |  |  |  |  |  | -.24 | -.25 |
| **14.** Emotional Exhaustion | 2.18  (1.04) | 1.00-  6.00 |  |  |  |  |  |  |  |  |  |  |  |  |  | .72** |
| **15.** Turnover Intentions | 1.97  (1.12) | 1.00-  5.00 |  |  |  |  |  |  |  |  |  |  |  |  |  |  |

*Note:* ***p* < .01 **p* <.05

**Table 3**

*Progress through the Implementation Guide by Team*

|  | **TeamTRACS Team 1** |  | **TeamTRACS Team 2** |  | **TeamTRACS Team 3** |  | **TeamTRACS Team 4** |
| --- | --- | --- | --- | --- | --- | --- | --- |
| *Exploration (Set the Stage)* |  |  |  |  |  |  |  |
| **Step 1:** Create a change team | Completed  Month 2 |  | Completed  Month 2 |  | Partial  Months 1-4 |  | Partial  Month 2 |
| **Step 2:** Look at the data | Completed  Month 2 |  | Completed  Months 2-3 |  | Partial  Month 3 |  | Completed  Month 2 |
| **Step 3:** Decide if team training is the right choice | Completed  Month 2 |  | Completed  Month 2 |  | Completed  Month 2 |  | Completed  Month 2 |
| *Preparation (Make a Plan)* |  |  |  |  |  |  |  |
| **Step 4:** Make an action plan for training | Completed  Month 2 |  | Completed  Months 2-4 |  | Completed  Months 2-3 |  | Completed  Months 3-4 |
| *Implementation (Make it Happen)* |  |  |  |  |  |  |  |
| **Step 5:** Conduct the training | Completed  Months 3-4 |  | Completed  Months 4-5 |  | Completed  Month 5 |  | Completed  Month 4 |
| **Step 6:** Clarify strengths, challenges, and goals | Completed  Months 4-5 |  | Partial  Months 2-5 |  | Partial  Month 6 |  | Partial  Month unknown |
| **Step 7:** Encourage change | Completed  Months 4-7 |  | Partial  Months 6-7 |  | Partial  Months 6-8 |  | Partial  Months 7-8 |
| **Step 8:** Evaluate improvement | Completed  Months 6-7 |  | Not started |  | Not started |  | Partial  Month 8 |
| *Sustainment (Make It Stick)* |  |  |  |  |  |  |  |
| **Step 9:** Integrate changes | Completed  Months 6-7 |  | Not started |  | Not started |  | Not started |
| **Step 10:** Plan for continuous improvement | Completed  Month 7 |  | Not started |  | Not started |  | Not started |
| **Steps Completed** | 100% |  | 50% |  | 30% |  | 40% |
| **Steps Partially Completed** | 0% |  | 20% |  | 40% |  | 30% |

Trial Dates: July 2022 (Month 0) to February 2023 (Month 7).

The implementation guide is available upon request from the first author.

**Figure 1**

*Participant Flow Diagram*

CACs expressing interest (n = 12)

CACs invited to participate (n = 7)

CACs agreeing to participate (n = 6)

CACs randomized to TeamTRACS

(n = 4)

CACs randomized to waitlist comparison

(n = 2)

Eligible participants

(n = 130)

Completed baseline

(n = 87)

Eligible participants

(n = 136)

-22 individuals who left team

+28 individuals who joined team

Follow-Up

Completed follow-up

(n = 89)

Baseline data only (n = 28)

Baseline and follow-up data (n = 59)

Follow-up data only (n = 30)

Enrollment

Allocation

Baseline

Analyses

Eligible participants

(n = 67)

Completed baseline

(n = 48)

Eligible participants

(n = 66)

-10 individuals who left team

+9 individuals who joined team

Completed follow-up

(n = 40)

Baseline data only (n = 15)

Baseline and follow-up data (n = 33)

Follow-up data only (n = 7)

CAC: Children’s Advocacy Center

**Figure 2**

*Participant Disciplines*

*Note:* Accreditation standards require multidisciplinary teams to include seven core disciplines: Law enforcement, mental health, child protective services, victim advocacy, Children’s Advocacy Center, medical, and prosecution. Individuals may fill multiple roles (e.g., CAC director and forensic interviewer). Four of six teams had participation from all core disciplines. One team was missing medical representation (did not include a medical provider on their roster), and one team did not have participation from prosecution.

**Figure 3**

*Psychological Safety: Marginal Effects of Intervention Condition and Timepoint*


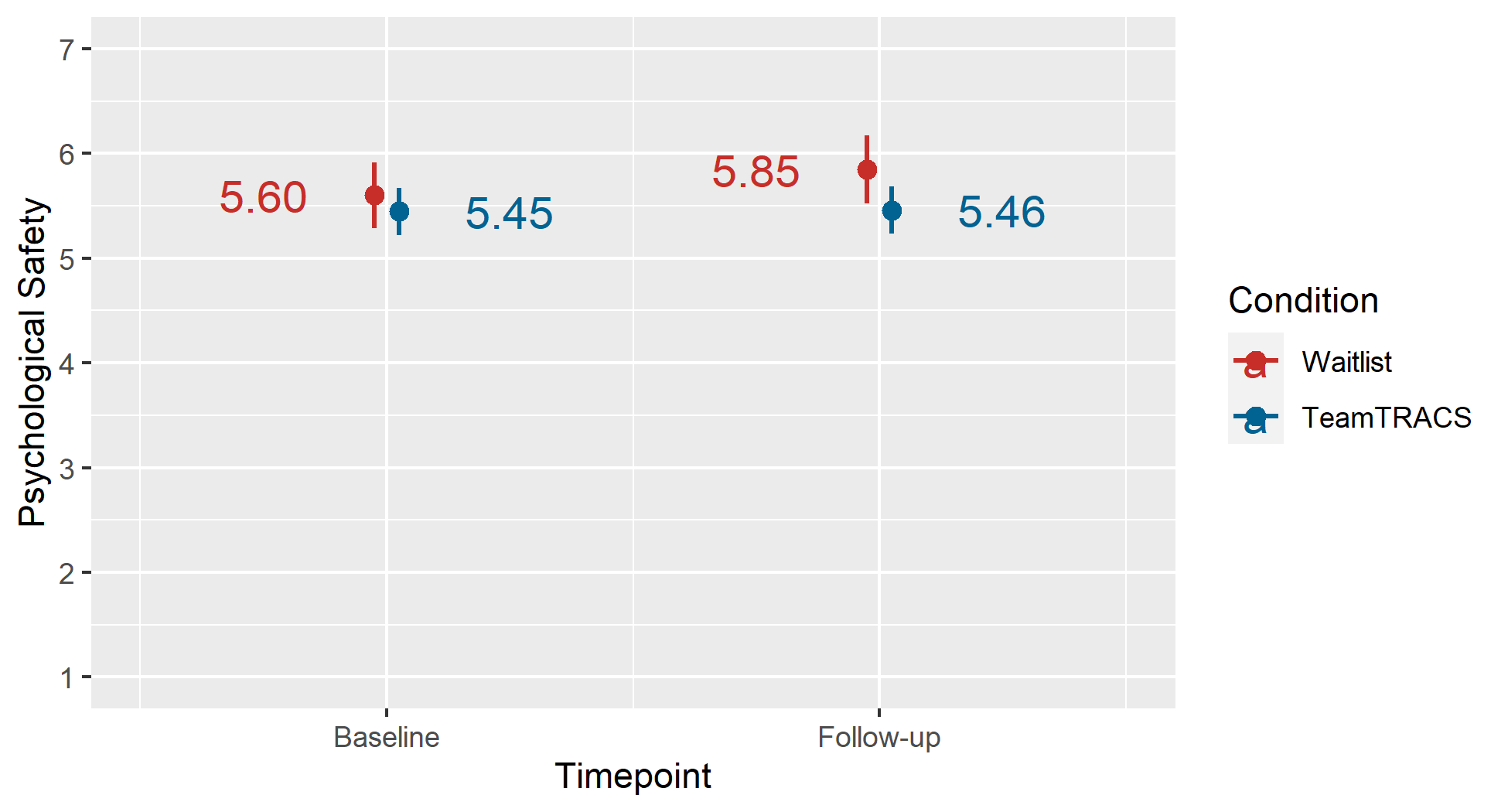


**Figure 4**

*Learning Behavior: Marginal Effects of Intervention Condition and Timepoint*


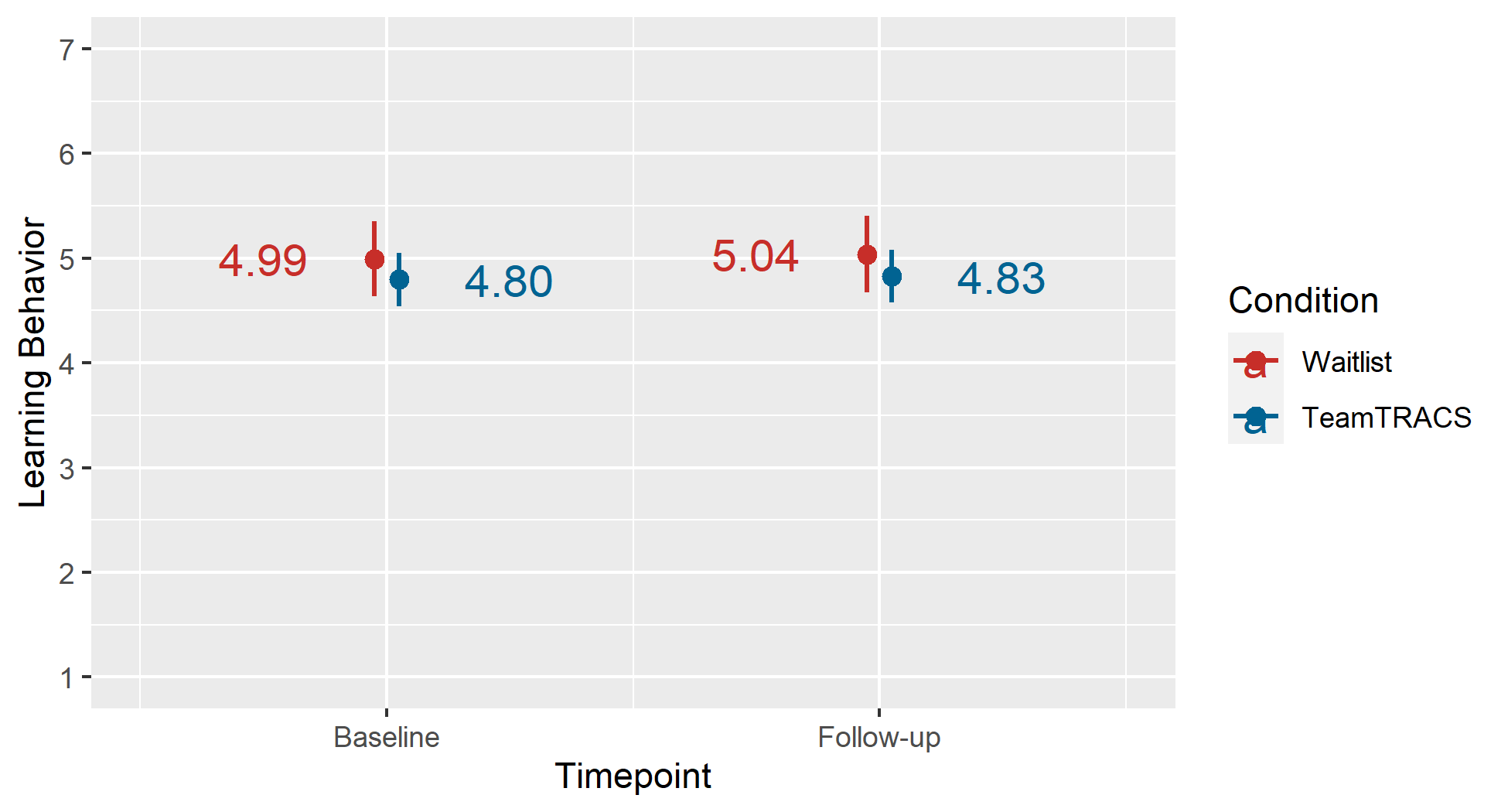


**Figure 5**

*Team Performance: Marginal Effects of Intervention Condition and Timepoint*


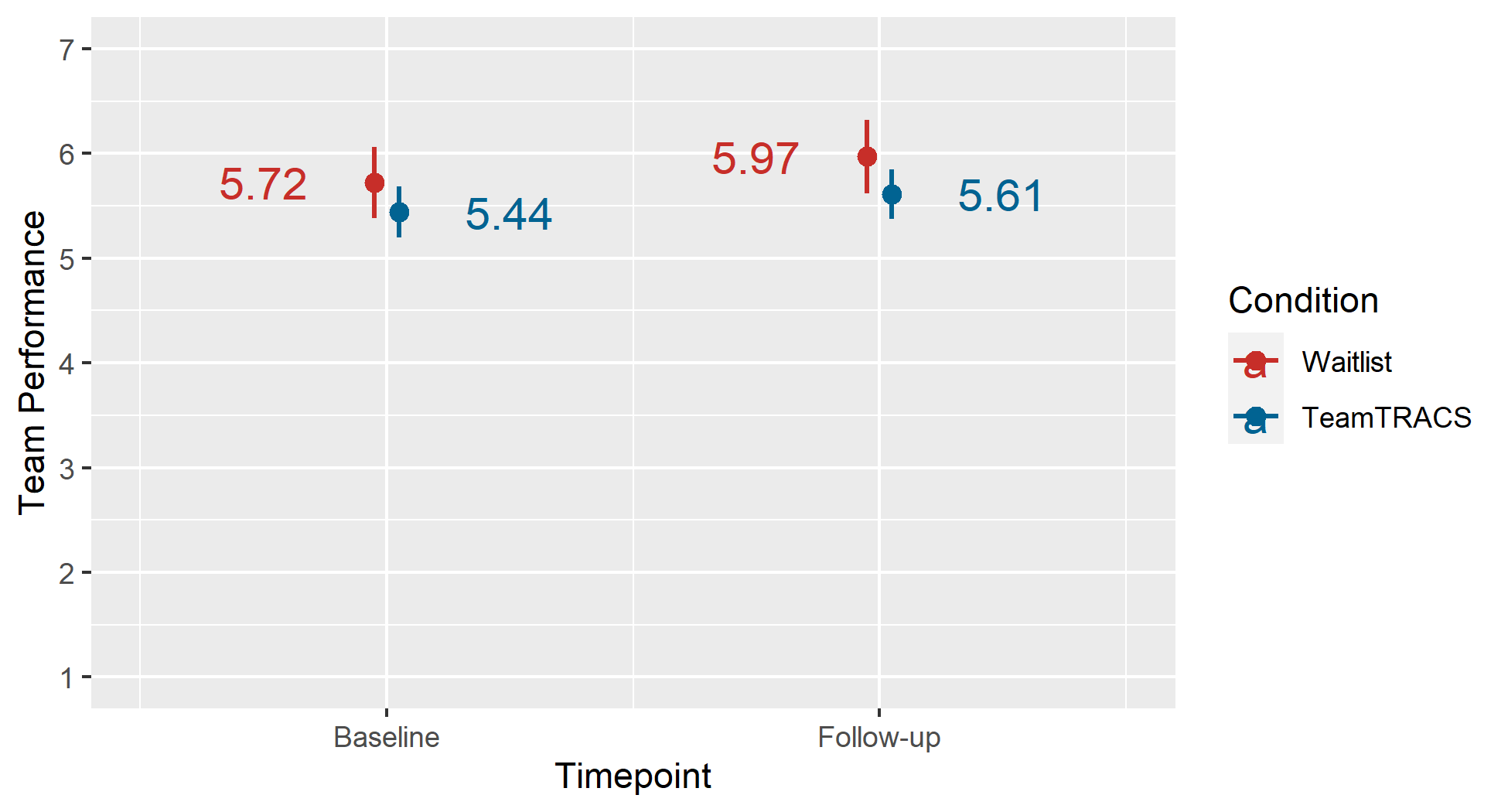


**Figure 6**

*Team Roles and Respect: Average Team Scores at Baseline and Follow-up*


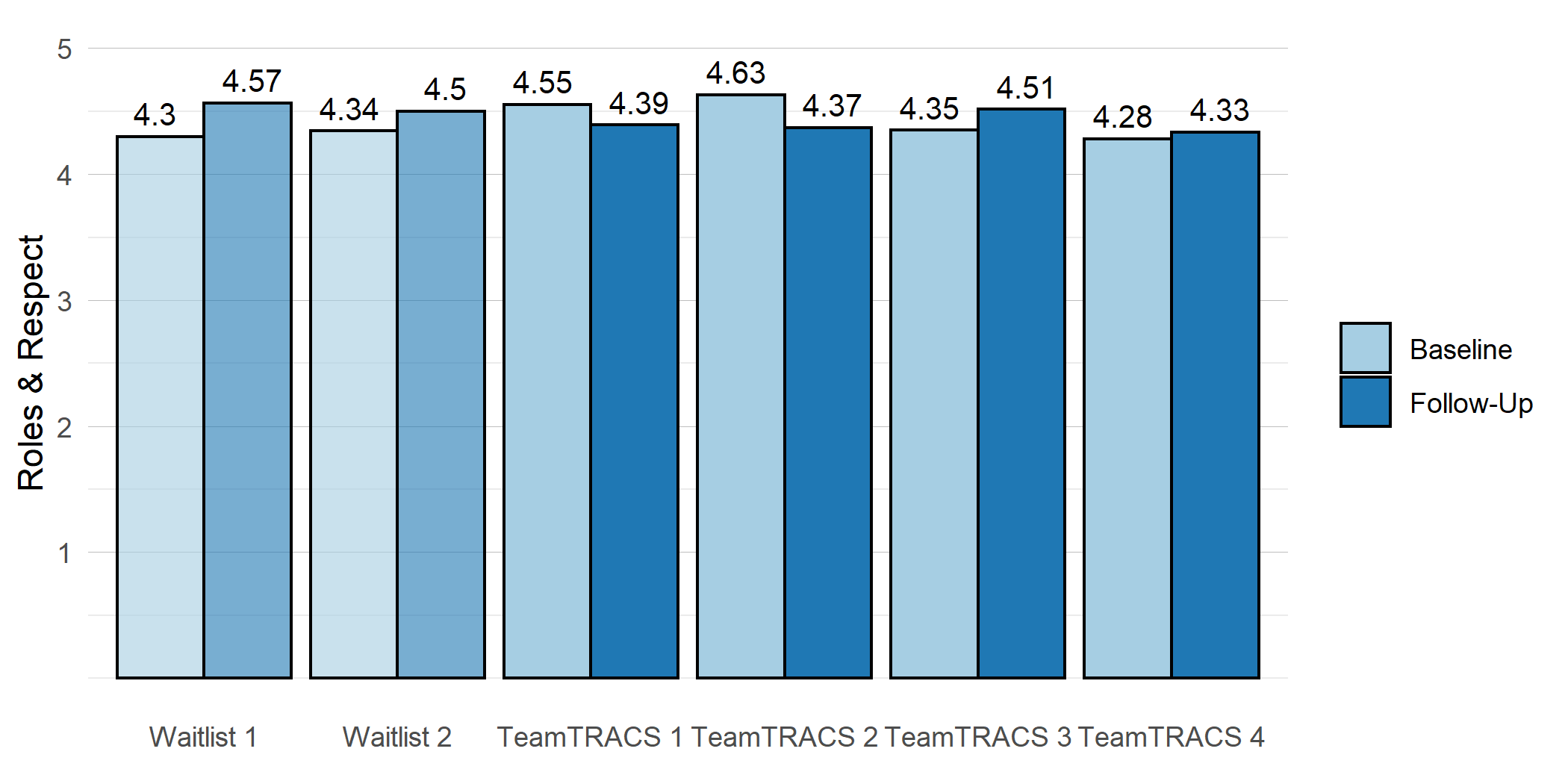


**Figure 7**

*Shared Awareness: Average Team Scores at Baseline and Follow-up*


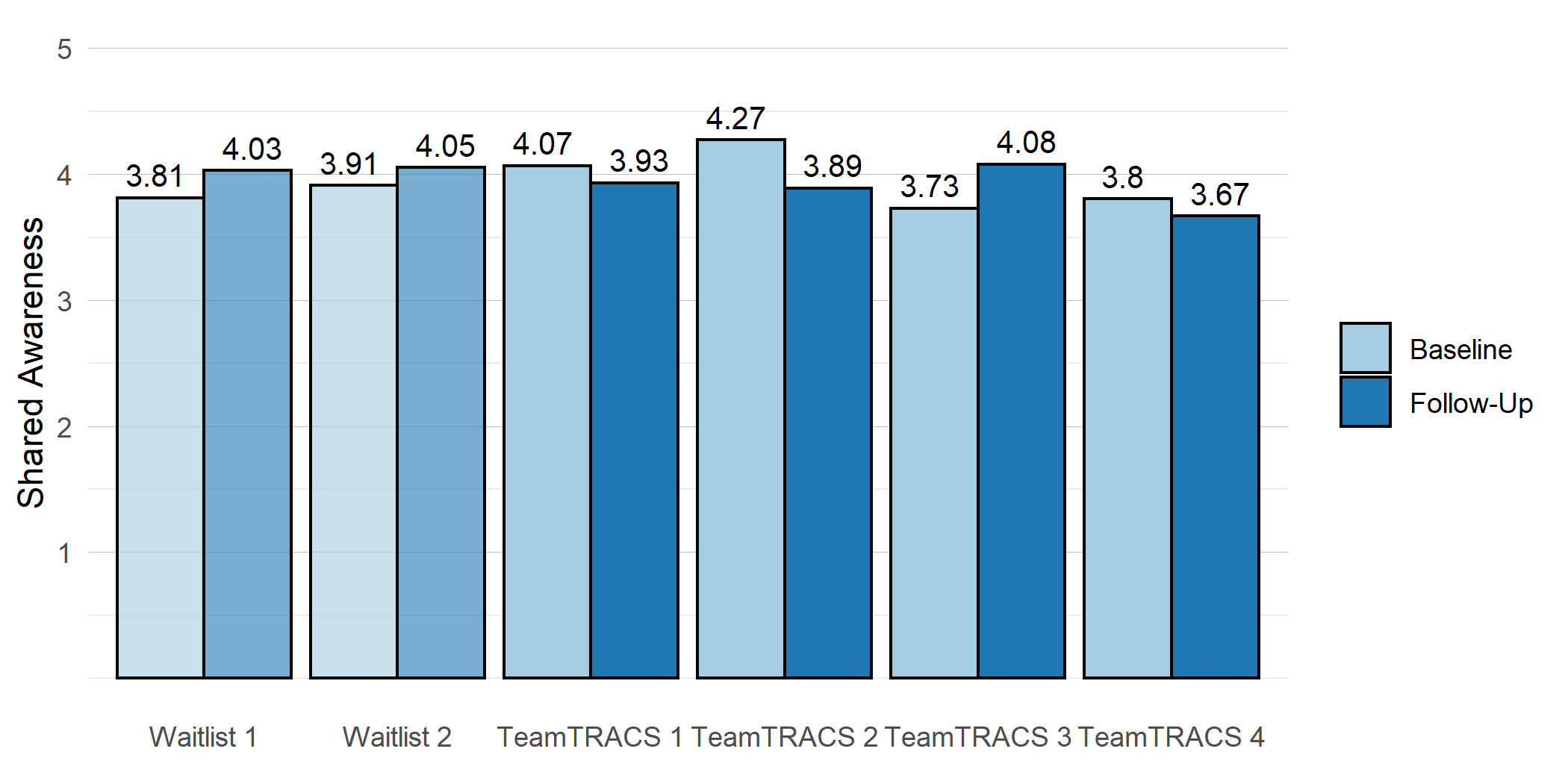


**Figure 8**

*Information Exchange: Average Team Scores at Baseline and Follow-up*


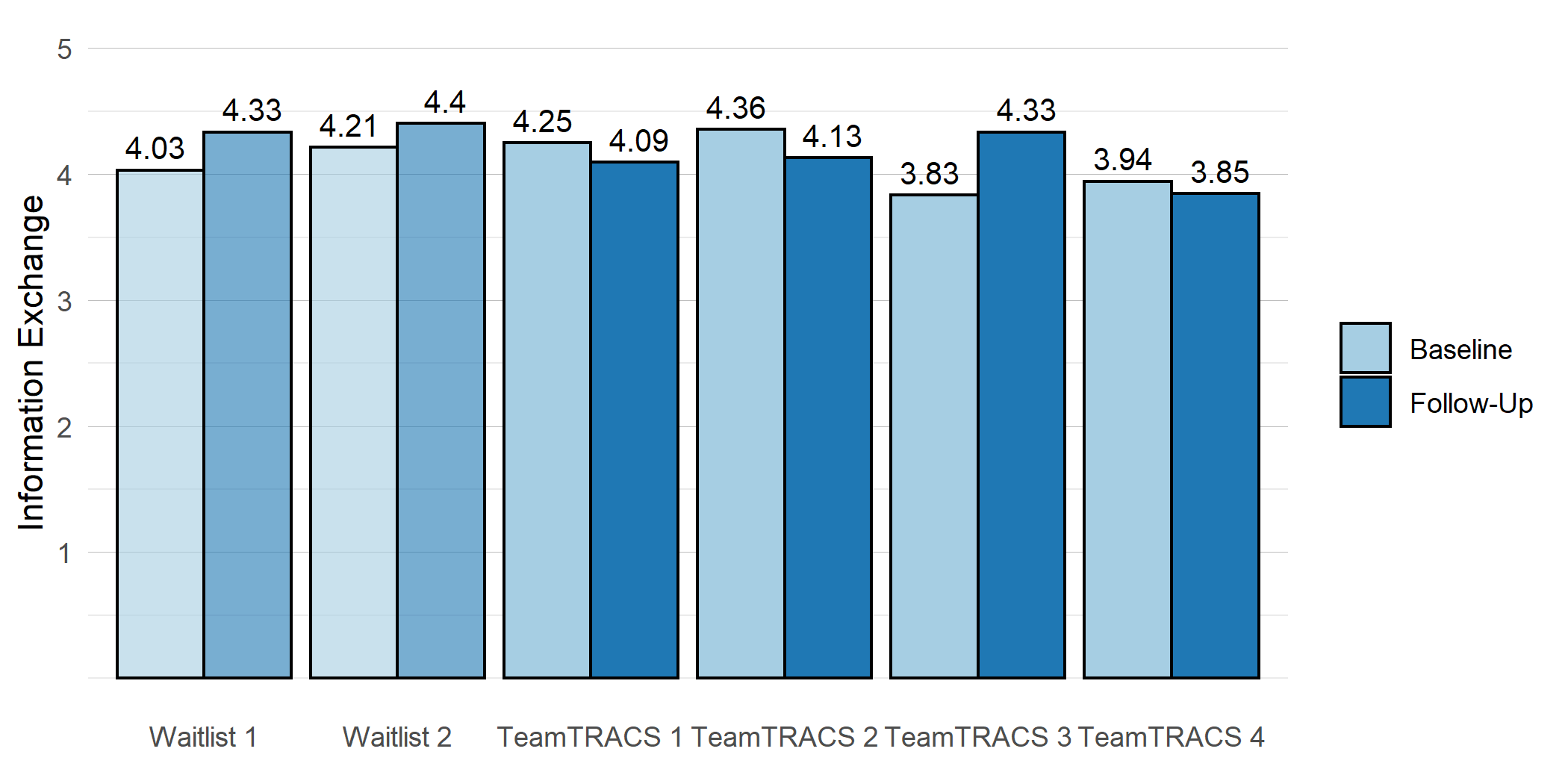


**Figure 9**

*Conflict Management: Average Team Scores at Baseline and Follow-up*


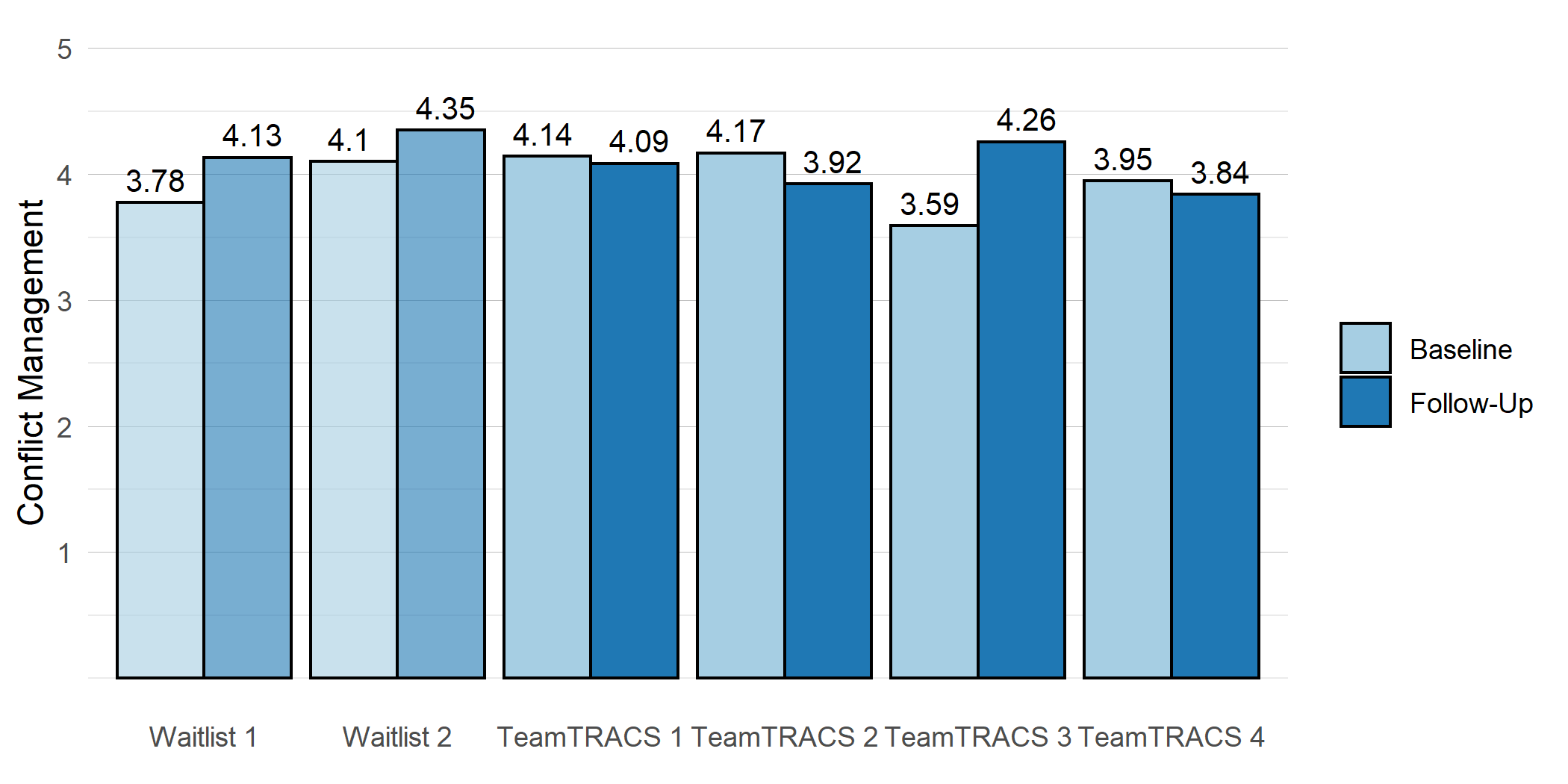


**Figure 10**

*Supportive Behavior: Average Team Scores at Baseline and Follow-up*


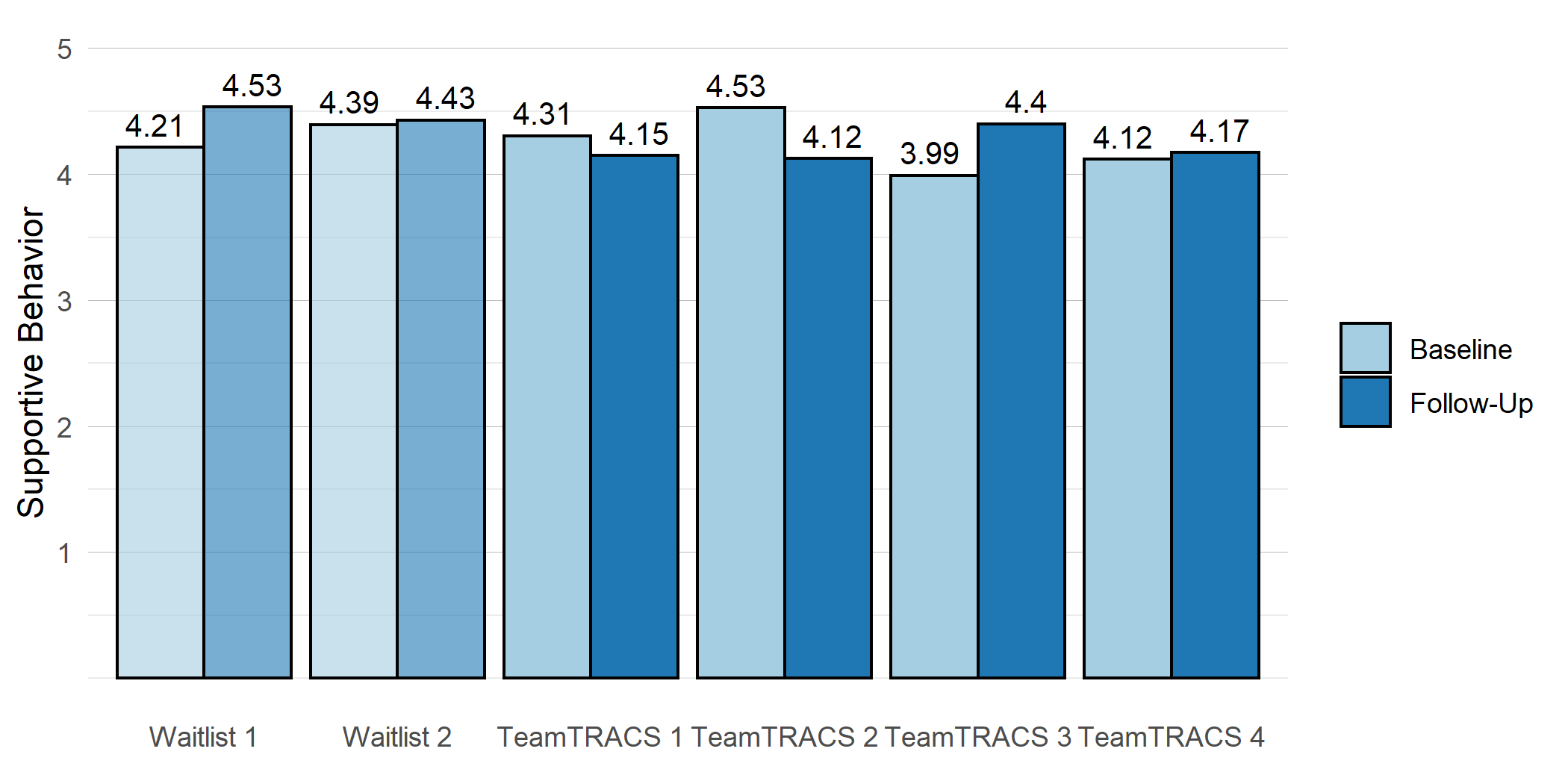


**Figure 11**

*Reflexivity: Average Team Scores at Baseline and Follow-up*


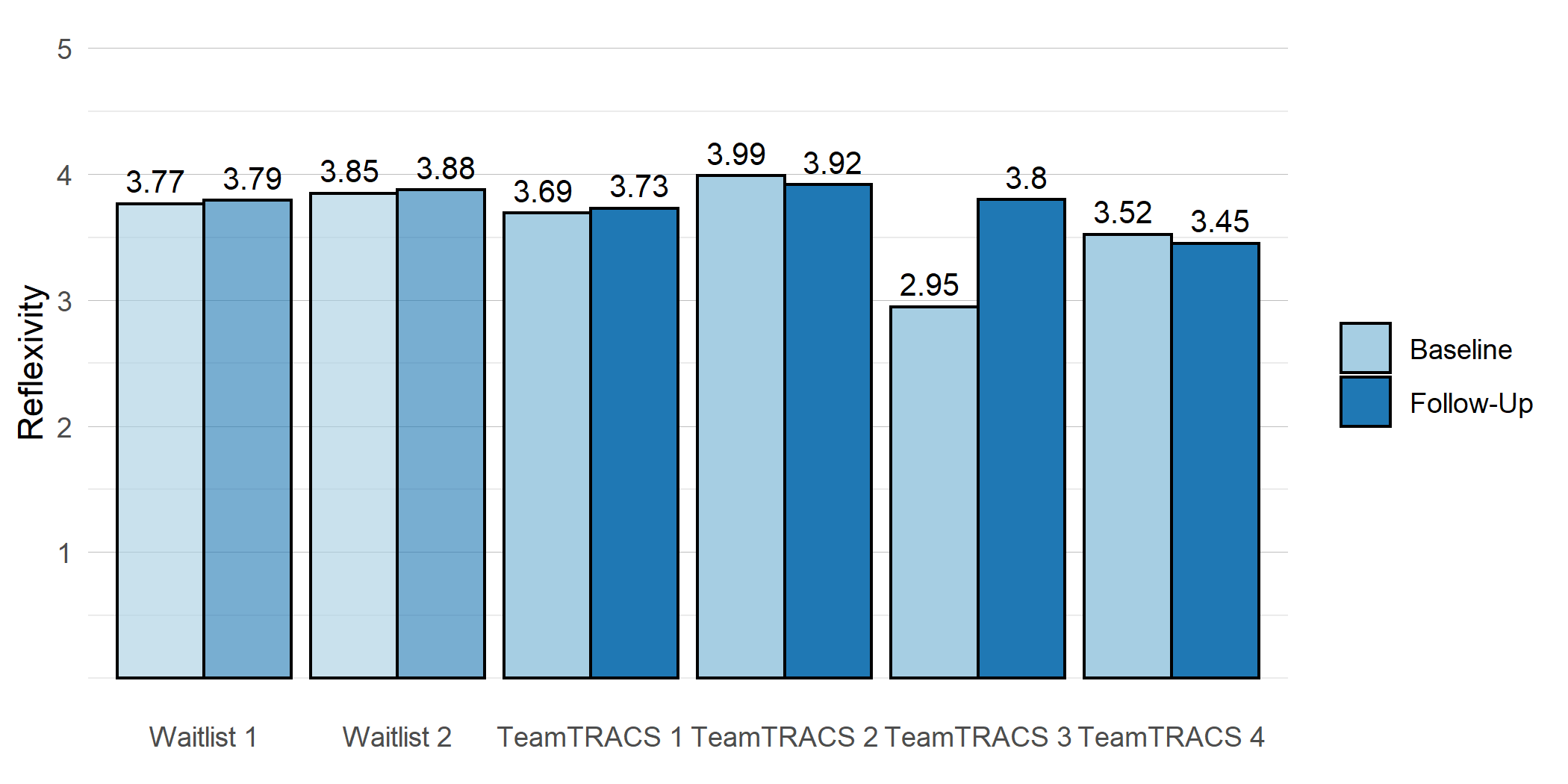


**Figure 12**

*Clear Direction: Average Team Scores at Baseline and Follow-up*


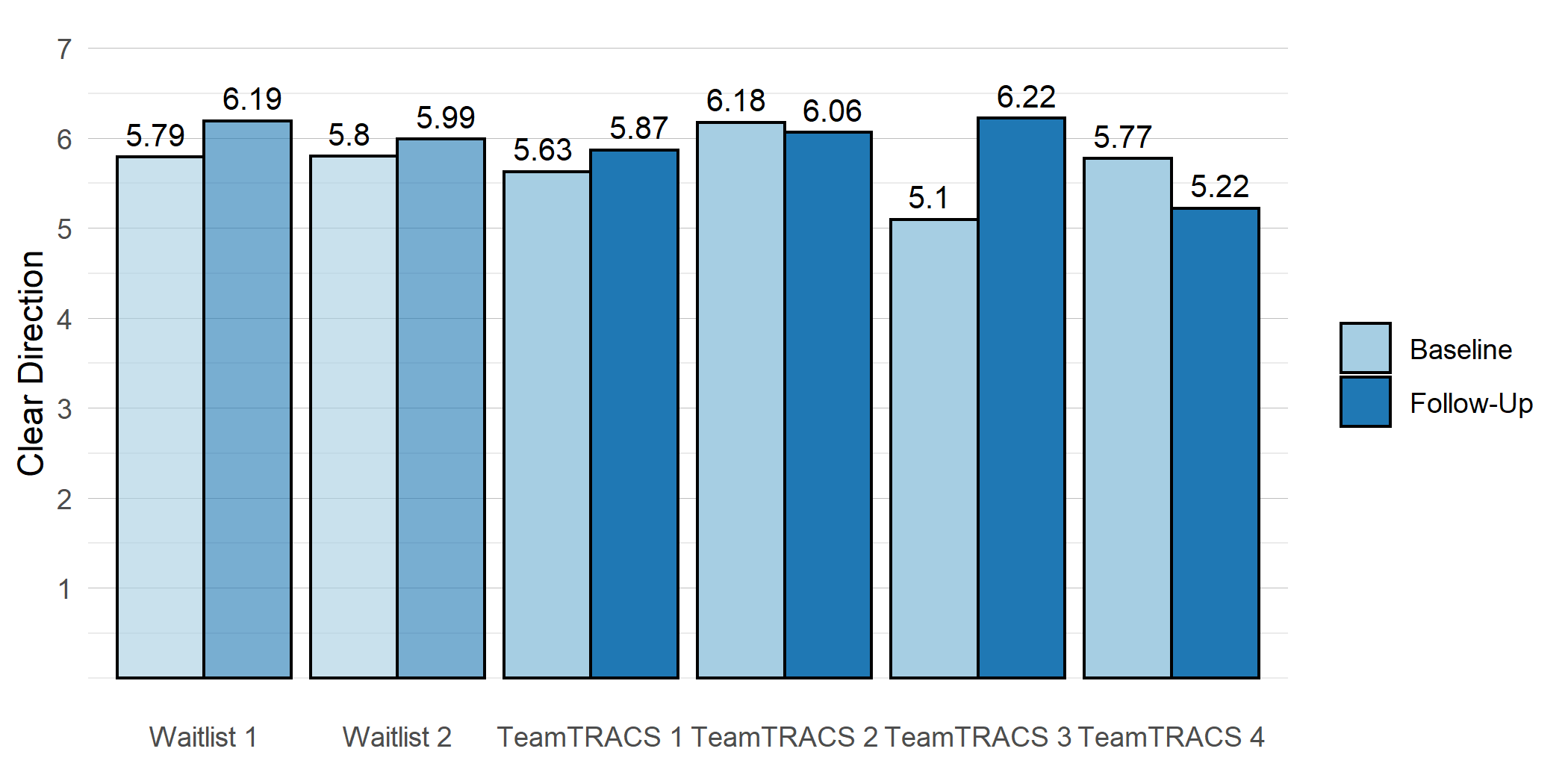


**Figure 13**

*Work Engagement: Marginal Effects of Intervention Condition and Timepoint*

*
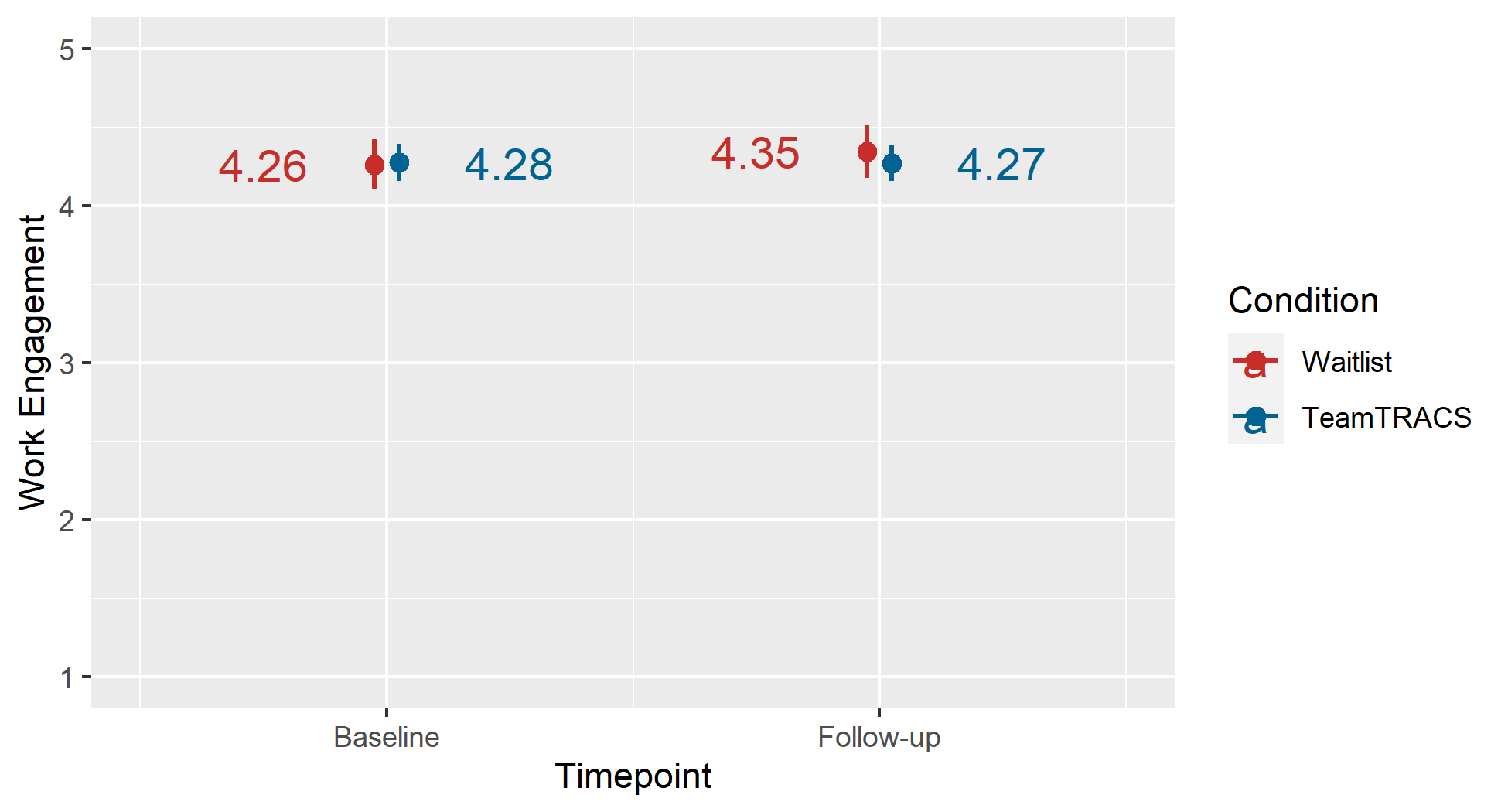
***Figure 14**

*Emotional Exhaustion: Marginal Effects of Intervention Condition and Timepoint*

*
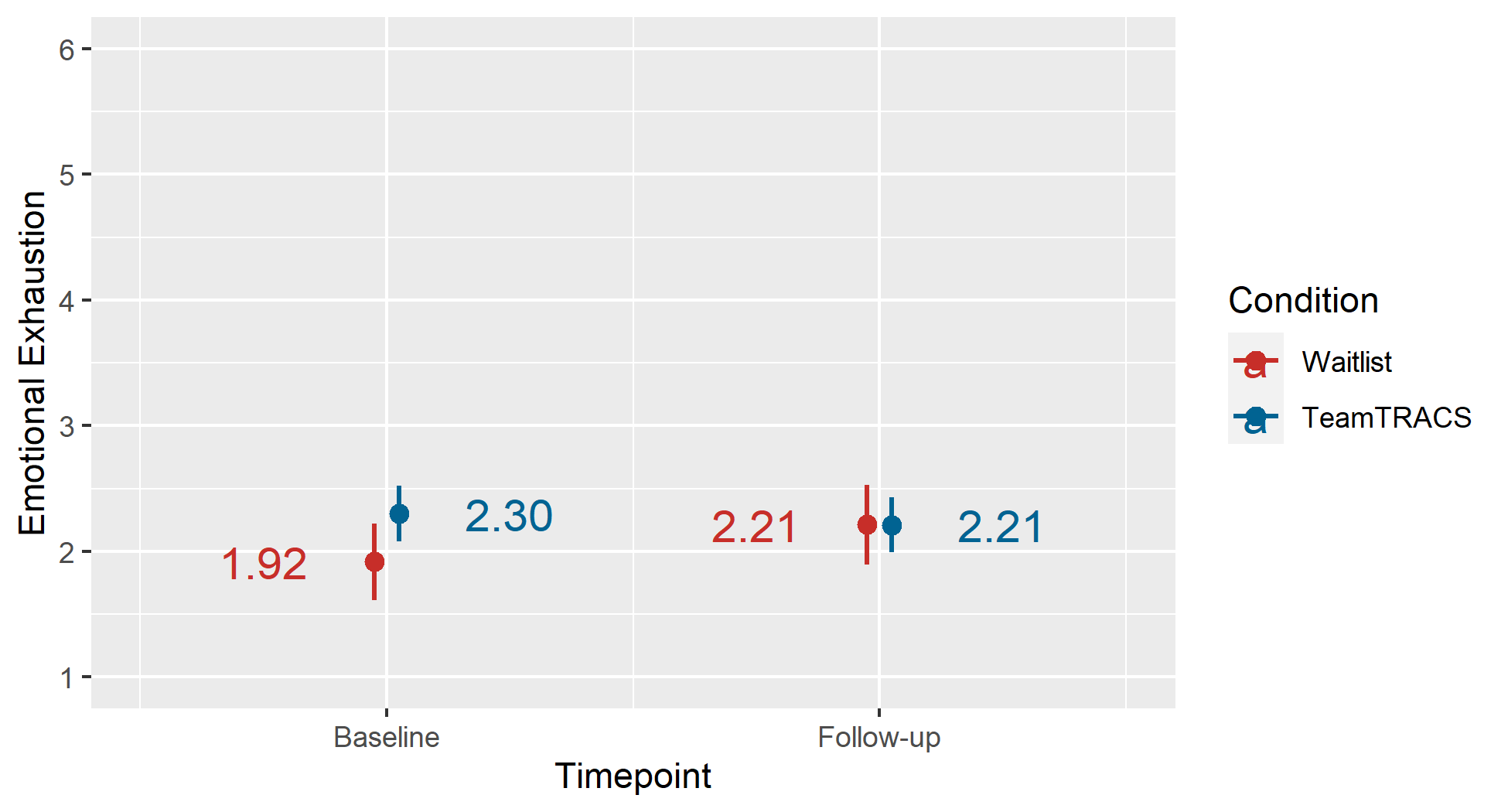
*

**Figure 15**

*Turnover Intentions: Marginal Effects of Intervention Condition and Timepoint*

*
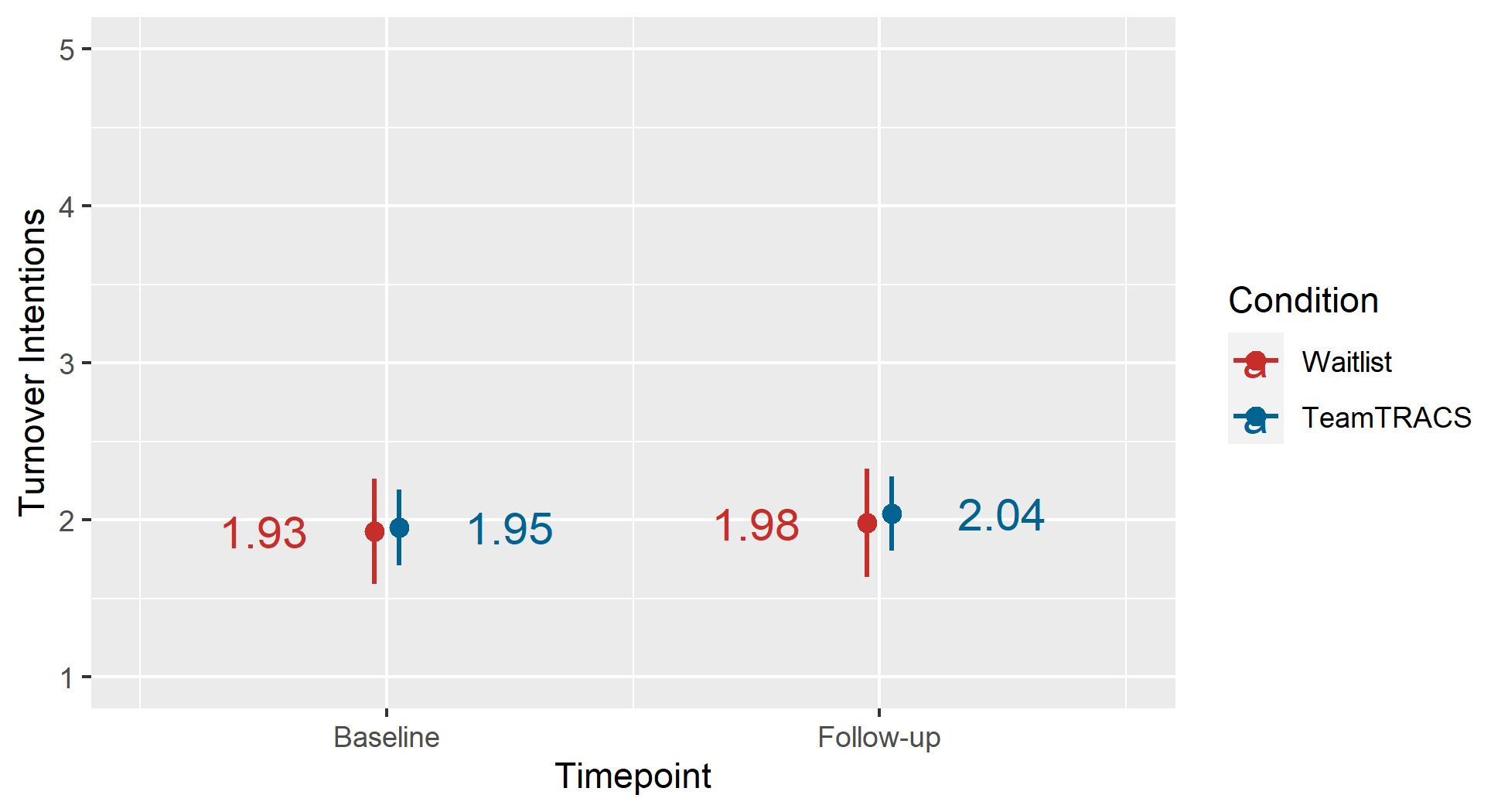
*

**Supplemental Material – Summaries of Implementation Progress by Team**

**TeamTRACS Team 1**

In TeamTRACS Team 1, the program manager was able to easily assemble a change team. Change team members were invested and engaged, and the manager was very organized. They set a recurring meeting time and met regularly before case review meetings for at least 6 months. The change team used the guide and worksheets, often completing worksheets on their own for brainstorming and then working together to create a final version. The change team moved quickly to determine the details of the training workshop and successfully held an in-person training workshop. Attendance was good, and MDT members were engaged. After the training, the change team set their goals and took actions. They were able to achieve their goal (hold a full MDT training) in February, 3 months ahead of their planned timeline. After achieving this goal, they identified a new goal and made plans for continuous improvement, including identifying how they will evaluate the MDT and when they will review and revise goals and procedures. The change team was motivated and met regularly, which helped them work through the implementation guide. All steps were completed during the 7-month period.

**TeamTRACS Team 2**

In TeamTRACS Team 2, the MDT coordinator created and led a change team. They discussed the strengths and challenges of MDT and set goals together. The team went into the training workshop with good momentum, and team members enjoyed the workshop. However, after the workshop, the implementation of TeamTRACS fell off track because of changes in staffing and leadership, including the MDT coordinator going on leave unexpectedly, turnover and promotions at the CAC, and high turnover at partnering agencies. There is still interest in TeamTRACS but team members are less engaged than they were initially. The MDT coordinator used the implementation guide initially and had positive feedback. Her replacement has not used the guide but is interested in doing so and re-engaging the team in TeamTRACS. In the TeamTRACS Implementation Guide, Steps 1-5 were completed and there were some efforts related to Steps 6 & 7. Steps 8-10 were not started.

**TeamTRACS Team 3**

TeamTRACS Team 3 was struggling with very low engagement at the start of the study. The MDT Coordinator reported feeling frustrated at the lack of engagement and unable to interest others in improving the team. No change team was ever created. The MDT Coordinator took responsibility for implementation and did the work herself with some input and support from CAC leadership. Although it was unclear if the team was ready for team training or would participate in the study, the MDT Coordinator decided to move forward with training. Attendance at the training was better than expected, with representation from all disciplines. Team members were engaged in the training and gave honest feedback. The team continued to discuss their goals after the training and exhibited substantial improvement in their engagement and willingness to discuss how the team is working. No specific plans or steps were taken to encourage change or evaluate improvements other than consistently and persistently showing a willingness to discuss and make changes, which has facilitated progress. The team continued to improve after the study ended. The MDT Coordinator reported noticeable improvements in case review participation, communication, and psychological safety. In the TeamTRACS Implementation Guide, Steps 3-5 were completed and there were some efforts related to Steps 1, 2, 6, & 7. Steps 8-10 were not started.

**TeamTRACS Team 4**

In TeamTRACS Team 4, the MDT Coordinator was able to gather some team members who were interested in discussing the team, although only one discipline was involved. After an initial discussion of team strengths and weaknesses, the change team did not meet again. The training had lower participation than expected. Some individuals who were not expected to attend came, while others who were expected did not. Those who attended seemed to enjoy the training. However, there was still little buy-in from team members, with responsibility for implementing TeamTRACS left to the CAC. Additional challenges included turnover and agency leadership changes. The MDT Coordinator was promoted and hiring a replacement took several months. There was little progress immediately after the training workshop. At the final reflection, after the end of the study, some changes and improvements were reported. The [former] MDT Coordinator and supervisor reviewed feedback from the training and began making small changes to meetings and taking actions to strengthen relationships with team members. These changes had a positive impact, resulting in stronger relationships, better communication between law enforcement and child welfare outside of meetings, and more frequent direct communication between team members. Team members have been more vocal and willing to provide feedback. They received positive feedback on these change from the team through a biennial survey and plan to continue making small changes over time. They will be rewriting protocols soon and hope to involve leadership from different agencies in the process to increase their investment. In the TeamTRACS Implementation Guide, Steps 2-5 were completed and there were some efforts related to Steps 1, 6, 7, & 8. Steps 9-10 were not started. Progress on Steps 6-8 was mostly made after the end of the study (Months 7-8).
